# Cobalt Oxide 2D Nanosheets Formed at a Polarized Liquid|Liquid Interface toward High-Performance Li-Ion and Na-Ion Battery Anodes

**DOI:** 10.1021/acsami.3c11795

**Published:** 2023-12-05

**Authors:** Bharathi Konkena, Chakrapani Kalapu, Harneet Kaur, Angelika Holzinger, Hugh Geaney, Valeria Nicolosi, Micheál D. Scanlon, Jonathan N. Coleman

**Affiliations:** † School of Physics, CRANN & AMBER Research Centres, Trinity College Dublin, Dublin D2 D02 K8N4, Ireland; ‡ Micro Nano Systems Department, Tyndall National Institute, Cork T12 R5CP, Ireland; § The Bernal Institute and Department of Chemical Sciences, University of Limerick, Limerick V94 T9PX, Ireland; ∥ School of Chemistry, CRANN & AMBER Research Centres, Trinity College Dublin, Dublin D2 D02 W9K7, Ireland

**Keywords:** liquid/liquid interface, cobalt oxide, two-dimensional platelets, Li-ion batteries, Na-ion batteries, rate performance

## Abstract

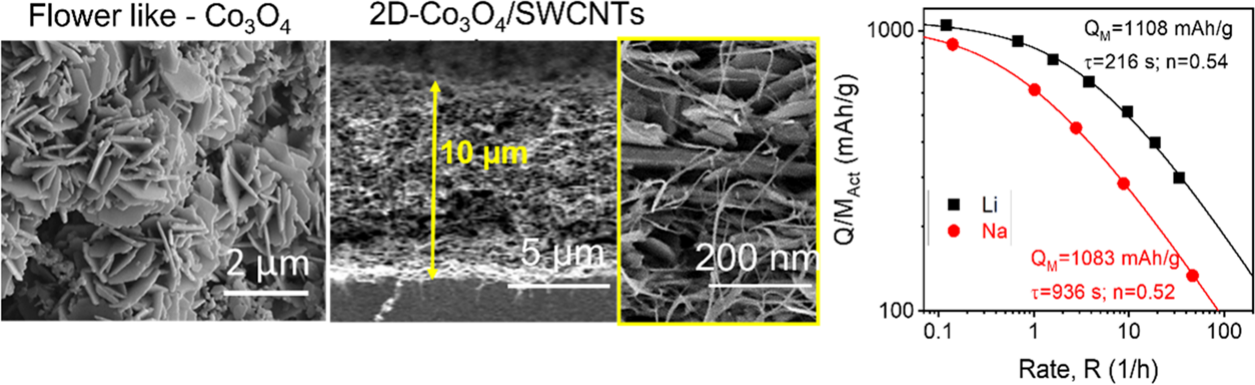

Cobalt oxide (Co_3_O_4_)-based nanostructures have the potential as low-cost materials for lithium-ion (Li-ion) and sodium-ion (Na-ion) battery anodes with a theoretical capacity of 890 mAh/g. Here, we demonstrate a novel method for the production of Co_3_O_4_ nanoplatelets. This involves the growth of flower-like cobalt oxyhydroxide (CoOOH) nanostructures at a polarized liquid|liquid interface, followed by conversion to flower-like Co_3_O_4_ via calcination. Finally, sonication is used to break up the flower-like Co_3_O_4_ nanostructures into two-dimensional (2D) nanoplatelets with lateral sizes of 20–100 nm. Nanoplatelets of Co_3_O_4_ can be easily mixed with carbon nanotubes to create nanocomposite anodes, which can be used for Li-ion and Na-ion battery anodes without any additional binder or conductive additive. The resultant electrodes display impressive low-rate capacities (at 125 mA/g) of 1108 and 1083 mAh/g, for Li-ion and Na-ion anodes, respectively, and stable cycling ability over >200 cycles. Detailed quantitative rate analysis clearly shows that Li-ion-storing anodes charge roughly five times faster than Na-ion-storing anodes.

## Introduction

Lithium (Li)-ion batteries (LIBs) are crucial for the functioning of portable electronic devices and the continued success of the electric vehicle revolution.^1,2^ However, the limited supply of Li has driven research into a range of alternative technologies, with perhaps the most promising being the storage of sodium (Na) ions.^3−5^ As the chemistries of Li and Na ions are similar, the working principles of sodium-ion batteries (SIBs) closely resemble those of LIBs.^6,7^ To maximize their impact, breakthroughs in the development of environmentally friendly electrode materials for use in both technologies are required.

Among many anode materials, transition metal oxides (TMOs) are widely considered to be ideal electrode materials for both LIBs and SIBs, due to their significant theoretical specific capacities which range from 600 to 1100 mAh/g.^8−11^ Of the many TMOs, cobalt oxide (Co_3_O_4_), with an inverse spinel structure, is attractive because of its relatively low cost and impressive theoretical capacity (890 mAh/g).^12^ However, metal oxides, such as Co_3_O_4_, often suffer from poor electronic conductivity and large volume expansion, which limits their performance.^12,13^

To date, there have been considerable attempts to maximize the electrochemical efficiency of Co_3_O_4_ anodes, usually by synthesizing nanostructured materials with various morphologies, such as one-dimensional nanotubes,^14^ nanoneedles,^15^ nanowires,^16^ nanocages,^17^ two-dimensional (2D) nanosheets,^18^ and three-dimensional (3D) nanocubes.^19^ In particular, 2D nanoplatelets of Co_3_O_4_ with mesoporous structure, high specific surface area, and short solid-state diffusion paths are particularly promising. However, in all cases, the synthetic methods employed typically include complex steps, often requiring high temperatures, templates, or solid supports, and are expensive to implement. A useful alternative is the electrosynthesis^20,21^ or self-assembly^22^ of well-defined 2D nanostructures in a controllable manner at an interface between two immiscible electrolyte solutions (ITIES), *i.e*., a polarized aqueous|organic or “soft” interface such as that formed between aqueous and 1,2-dichloroethane (DCE) electrolyte solutions. Such a polarized liquid|liquid interface creates a defect-free “2D confined space” that allows for the electrosynthesis or self-assembly of 2D nanosheets in a single step with excellent reproducibility.^22,23^ This method has become strongly established because of its potential applications in energy conversion and storage technologies.^24,25^ Recently, we reported a process to produce 2D nanoplatelets of α-Fe_2_O_3_: growth of α-Fe_2_O_3_ nanoflowers at a polarized aqueous interface, followed by sonication-induced conversion into 2D nanoplatelets. The α-Fe_2_O_3_ 2D nanoplatelets were then combined with carbon nanotubes to develop LIB anodes that deliver outstanding anode performance with low-rate capacities nearly reaching 1500 mAh/g (competing with the best literature results).^26^ After an extended activation process, a remarkable enhancement in low-rate capacities was observed, which surpassed 2000 mAh/g (after 345 cycles, the capacity stands at 2115 mAh/g).^26^

Methods that yield nanostructured active materials, such as using an ITIES, are useful in battery research as small active particles give porous electrodes with high capacity and excellent rate performance.^27−29^ In particular, using active particles in the form of 2D platelets has an advantage over spherical particles because they possess three times less surface area per volume for a given solid-state diffusion length.^28,30^ This gives short diffusion lengths, and so reduced solid-state diffusion times and enhanced rate performance,^31^ without extremely large active surface areas. In this manner, unwanted parasitic reactions are reduced between the active electrode materials and the nonaqueous electrolyte at different potentials.^32^

Another advantage of nanostructured materials is the ease with which charge can be delivered to all areas of the electrode using conductive additives.^33,34^ Co_3_O_4_ nanostructures have been incorporated into battery electrodes by mixing with a range of carbonaceous additives including graphene, carbon nanotubes, carbon nanofibers, and carbon black.^35^ One advantage of using carbon nanotubes is that in addition to enhancing the conductivity, they mechanically strengthen the electrode. This reinforcement allows the electrode to accommodate the expansion and contraction that occurs during lithiation/delithiation and sodiation/desodiation.^32,36^ In recent years, we have studied composites of a range of active materials mixed with carbon nanotubes using no additional binder/conductive additive.^26,32,36−38^ This type of electrode architecture allows us to produce LIB and SIB electrodes that almost always display near-theoretical capacities. In addition, this architecture enhances conductivity to deliver charge throughout the electrode by accommodating the expansion and contraction associated with lithiation/sodiation and delithiation/desodiation.

In this work, we prepare 2D nanoplatelets of Co_3_O_4_ in a three-step process. We first produce flower-like nanostructures consisting of CoOOH 2D nanosheets via interfacial synthesis at a polarized ITIES. These CoOOH nanostructures are converted by calcination into Co_3_O_4_ nanoflowers. Finally, these nanoflowers are converted into Co_3_O_4_ 2D nanoplatelets by sonication in a solvent. The resultant suspension of nanoplatelets can then be easily solution-processed. For example, solution mixing with carbon nanotubes allows the formation of composite suspensions which can easily be formed into nanotube/nanoplatelet composite films. Such films can be used as battery electrodes, with carbon nanotubes being used as both the binder and conductive additive. The resulting electrodes perform extremely well as both Li- and Na-storing anodes at a low current density (at 125 mA/g) in excess of 1000 mAh/g in each case. We performed a detailed quantitative rate analysis that revealed Li-ion storing anodes charge roughly five times faster than Na-ion storing anodes.

## Experimental Methods

All of the chemicals such as cobalt chloride (CoCl_2_), lithium perchlorate (LiClO_4_), 4-aminopyridine and tetrabutylammonium perchlorate (TBAClO_4_), and solvents *N*-methyl-2-pyrrolidone (NMP) and isopropanol (IPA) (HPLC grade >99%) were purchased from Sigma-Aldrich. P3-SWCNT was purchased from Carbon Solutions with carbonaceous purity >90%.

### Interfacial Synthesis of 3D Flower-like Structures Consisting of CoOOH 2D Nanosheets, Followed by Their Calcination to Form Co_3_O_4_ Nanostructures with the Same Morphology

**Figure 1:**
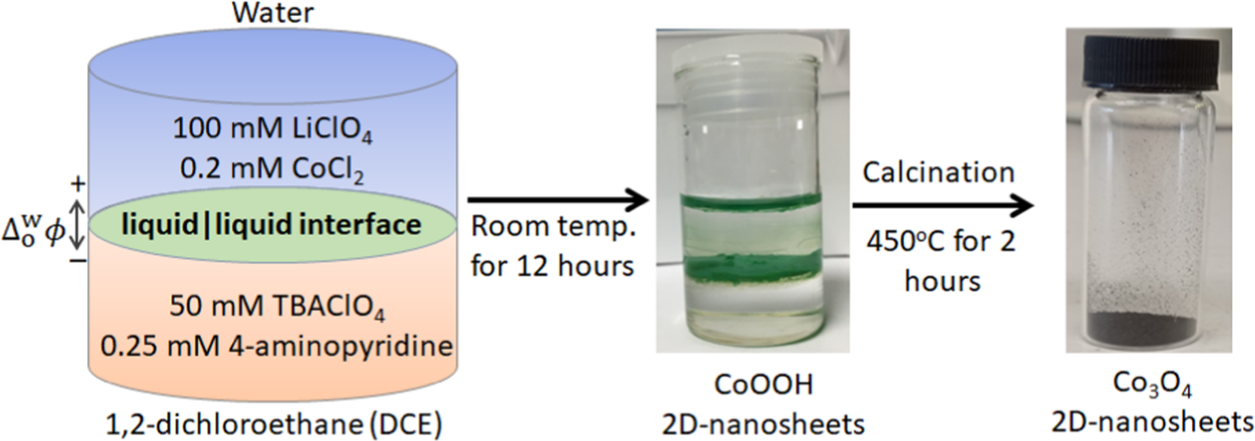
Schematic representation of the interfacial synthesis of 3D flower-like cobalt oxyhydroxide (CoOOH) structures composed of 2D nanosheets at a polarized aqueous|1,2-dichloroethane (DCE) interface and their subsequent conversion to cobalt oxide (Co_3_O_4_) 2D nanosheets after collection from the liquid|liquid interface and calcination.

Interfacial synthesis of CoOOH nanostructures was performed under ambient, aerobic conditions at an ITIES created between an acidic aqueous solution with 0.2 mM CoCl_2_ and 100 mM LiClO_4_ electrolyte, and an organic solution of DCE containing 0.25 mM 4-aminopyridine and 50 mM TBAClO_4_ electrolyte. Electrodeless polarization of the ITIES through the distribution of ClO_4_^–^ induces an applied Δ_o_^w^ϕ negative of the OCP recorded in the absence of polarization. This biphasic system was left for approximately 12 h at room temperature to allow the reactants sufficient time to diffuse toward the liquid|liquid interface and the interfacial complexation of CoCl_2_ with the 4-aminopyridine ligands to occur. Nucleation and growth of CoOOH particles occur and lead to the assembly of individual two-dimensional nanosheets into three-dimensional flower-like structures at the ITIES. This dark green material covers the whole aqueous|DCE interface (as shown in Figure 1). Afterward, the aqueous and organic phases were removed one after the other. The resulting precipitate was then collected and washed multiple times with a mixture of absolute ethanol and distilled water to ensure the effective removal of solvents. After thorough washing, the precipitate was dried at 80 °C overnight. Finally, the obtained powder was calcinated at 450 °C for 2 h, resulting in the production of black Co_3_O_4_ powder (Figure 1). To our knowledge, this is the first time Co_3_O_4_ nanoflowers have been made in this way (using an electrified liquid|liquid interface).

### Production of Co_3_O_4_ 2D Nanoplatelet Dispersion

Initially, 200 mg of the as-prepared Co_3_O_4_ powder was dispersed in 100 mL of isopropanol (IPA) in a metallic beaker. After dispersing, the solution was sonicated for 4 h using a tapered tip probe (VibraCell CVX, 750 W) with a 6 s on and 2 s off pulse, 45% amplitude, and under ice cooling. The obtained dispersion was centrifuged (Hettich Mikro 220R centrifuge with a fixed-angle rotor 1016) at 26*g* for 1 h. Next, the sediment with the large unexfoliated material was removed, while the supernatant with exfoliated sheets was further centrifuged at 2600*g* for 1 h. After discarding the resultant supernatant containing very small particles, we redispersed the sediment in 10 mL of IPA, resulting in a standard dispersion trapped between 26 and 2600*g* that consisted of polydisperse Co_3_O_4_ 2D nanoplatelets. To the best of our knowledge, this is the first report of making 2D nanoplatelets of Co_3_O_4_ directly from nanoflowers.

Powder X-ray diffraction (XRD) patterns were obtained using a Philips X’Pert Pro diffractometer with a Cu Ka radiation source (λ = 1.5418 Å) at 40 kV and 30 mA on the as-prepared powder samples. Zeiss Ultra Plus scanning electron microscope was used to capture scanning electron microscopy (SEM) images of the CoOOH powder, Co_3_O_4_, and Co_3_O_4_/SWCNT nanocomposite films. The images were taken by using an accelerating voltage of 2–5 kV and a 30 μm aperture at a working distance of 3–6 mm. Additionally, we examined the cross-sectional images of the fractured sides of the Co_3_O_4_/SWCNT composite films at room temperature.

Using the Horiba Jobin-Yvon LabRAM HR800, high-quality Raman spectra were acquired with a 633 nm excitation laser and a diffraction grating of 600 grooves, providing ∼1.5 cm^–1^ spectral resolution. Each spectrum is the average of 16 different spectra, ensuring consistent and reliable results. The measurements were carried out on both powdered CoOOH and thin films of Co_3_O_4_ dispersions obtained by drop-casting. To prepare for transmission electron microscopy (TEM) imaging, the standard dispersion of Co_3_O_4_ was drop-cast onto a holey carbon TEM grid and left to dry in air and finally dried overnight in a vacuum oven at 70 °C. Bright-field TEM imaging was performed using a JEOL 2100 microscope. In situ energy dispersive X-ray spectroscopy (EDX) spectroscopy was also performed by using an 80 mm^2^ XMAX EDX detector alongside TEM imaging. X-ray photoelectron spectroscopy (XPS) analysis of the Co_3_O_4_ was analyzed using a Thermo Scientific Multilab 2000 photoelectron spectrometer with a twin anode X-ray source.

### Electrochemical Characterization

To prepare the SWCNT dispersions with a concentration of 0.1 mg/mL, 8 mg of P3-SWCNT was added to 80 mL of isopropanol. The mixture was then sonicated using a horn-tip sonic probe (Vibracell CVX, 750 W) for 3 h, at 50% amplitude with an on/off pulse ratio of 6 s/2 s. To create Co_3_O_4_/SWCNT nanocomposite anodes, the Co_3_O_4_ dispersions were mixed with SWCNT dispersions in IPA. The mixture contains 80% Co_3_O_4_ and 20% SWCNT by weight. SWCNTs maximize both the mechanical stability and the electrical conductivity of the resulting films. No other additives were used. The next step was to vacuum-filter the mixture on a Celgard 2320 membrane with a thickness of 20 μm and an area of 2 cm^2^. For electrochemical testing, the composite films were cut into the required dimensions (*A* = 0.178 cm^2^). The areal mass loading of Co_3_O_4_ in the electrodes was approximately 1.0 mg cm^–2^, and with a thickness of 10 μm.

By using 2032-type coin cells (14 mm; MTI Corp.) in a glovebox with O_2_ and H_2_O contents lower than 0.1 ppm, half-cells were assembled and tested at room temperature. The counter/reference electrode used was a Li-metal disk, and a separator (Celgard C2320) with a thickness of 20 μm was used. Lithium hexafluorophosphate (LiPF_6_, 1.2 M in ethylene carbonate/ethyl methyl carbonate (EC/DMC, 1:1 in vol/vol, BASF) with 10 wt % fluoroethylene carbonate was used as the electrolyte for the Li-ion battery half-cell measurements. In Na-ion battery half-cells, fresh sodium metal was utilized as the counter and reference electrodes, with a glass fiber filter (Whatman GF 10) serving as a separator. The electrolyte consisted of sodium hexafluorophosphate (NaPF_6_, 1.2 M) in ethylene carbonate/dimethyl carbonate (EC/DEC, 1:1 in vol/vol, BASF), and it included 10 wt % fluoroethylene carbonates as an electrolyte additive.

The performance of the Co_3_O_4_/SWCNT nanocomposite anodes in Li-ion batteries and Na-ion batteries was thoroughly investigated with the help of a galvanostat-potentiostat (VMP-3, Biologic). Cyclic voltammetry was performed on the cells within a voltage range of 0.05 and 3.0 V versus Li^+^/Li for LIBs and 0.05 and 2.5 V versus Na^+^/Na for SIBs at multiple scan rates ranging from 0.1 to 1 mV/s. Additionally, the electrochemical properties of the anodes were measured with the galvanostatic charge–discharge mode within a voltage range of 0.05 and 3.0 V versus Li^+^/Li for LIBs and 0.05 and 2.5 V versus Na^+^/Na for SIBs, using an Arbin potentiostat.

## Results and Discussion

### Interfacial Synthesis and Characterization of α-Co_3_O_4_ 2D Nanosheets

#### Interfacial Synthesis

An aqueous|DCE interface can be polarized externally by using a potentiostat and specialized 4-electrode electrochemical cell, or chemically using an electrodeless approach by establishing a distribution interfacial Galvani potential difference (Δ_o_^w^ϕ) through partition of a common ion or salt between the aqueous and organic phases.^39−41^ Interfacial self-assembly of flower-like cobalt oxyhydroxide (CoOOH) nanosheets was achieved at a polarized aqueous|DCE interface using aqueous CoCl_2_ and organic soluble 4-aminopyridine ligand (Figures 1 and S1). This biphasic system consists of perchlorate (ClO_4_^–^) as a common anion. The supporting electrolyte salts in the aqueous and DCE phases are lithium perchlorate (LiClO_4_) and tetrabutylammonium perchlorate (TBAClO_4_), respectively. The Nernst–Donnan equation,^39,42^ states that the electrodeless polarization of the ITIES through the distribution of ClO_4_^–^ induces an applied Δ_o_^w^ϕ negative of the open circuit potential (OCP) recorded in the absence of polarization.

The ClO_4_^–^ anions may play a dual role in the interfacial self-assembly of CoOOH nanosheets. First, aqueous ClO_4_^–^ anions are relatively hydrophobic, *e.g*., by comparison with chloride (Cl^–^) anions, and can thus facilitate the transfer of reactants such as 4-aminopyridine from the organic phase (Figure 1). The latter promotes a higher driving force for interfacial complexation between CoCl_2_ and the 4-aminopyridine ligands. We propose that the resulting complex tunes the Co(III)/(II) redox potential such that Co^2+^ is oxidized to Co^3+^ by dissolved O_2_ (present in both the aqueous and organic phases). The process begins with oxidation to Co^3+^, which initiates the growth of CoOOH and leads to the self-assembly of individual flakes and particles at the aqueous|DCE interface (as depicted in Figure 1). Furthermore, negative polarization of the aqueous|DCE interface by distribution of ClO_4_^–^ may slow the kinetics of Co^2+^ interacting with the 4-aminopyridine at the interface. This creates suitable confined space for the growth of flower-like, as opposed to spherical, nanostructures. TBA^+^ cations are suggested to act as a structure-directing reagent, facilitating the morphological transition from 2D to 3D flower-like architectures.^26,44^ The green-colored CoOOH nanostructured material directly extracted from the liquid|liquid interface was subsequently converted to polycrystalline Co_3_O_4_ 2D nanosheets by calcination at 450 °C for 2 h, yielding a black powder as shown in Figure 1. This synthetic route allows for a simple fabrication of 2D nanostructures for electrochemical storage of specific alkaline ions, *i.e*., Li and Na.

#### Microscopic and Spectroscopic Characterization

**Figure 2:**
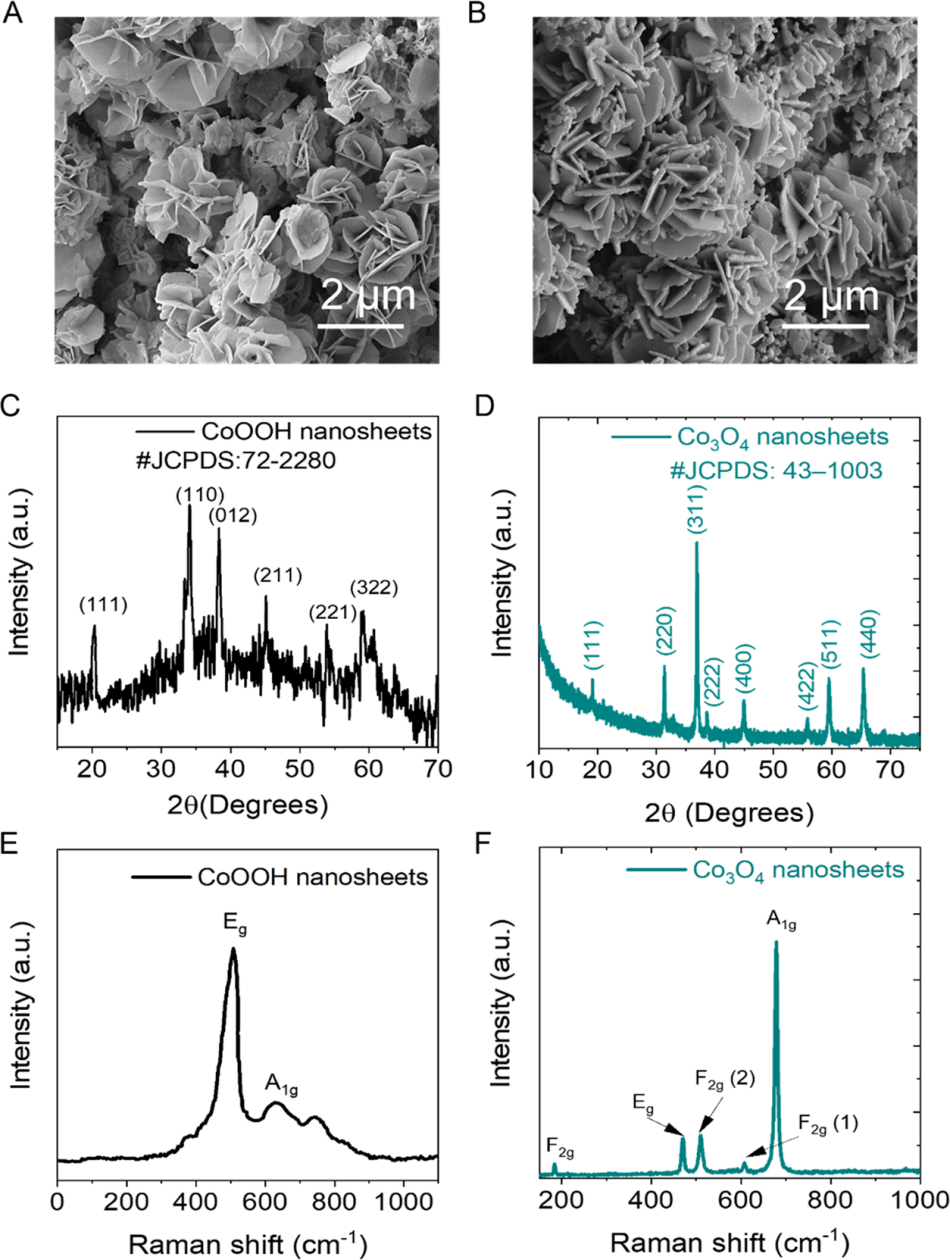
Structural characterization of the CoOOH and Co_3_O_4_ 3D flower-like nanostructures consisting of 2D nanosheets. SEM images of (A) CoOOH obtained directly after synthesis at the polarized liquid|liquid interface and (B) Co_3_O_4_ obtained after calcination of the interfacially grown CoOOH. X-ray diffraction patterns of (C) interfacially grown CoOOH and (D) Co_3_O_4_ obtained after calcination. Raman scattering spectrum of (E) interfacially grown CoOOH and (F) Co_3_O_4_ obtained after calcination.

As shown in Figure 2A, scanning electron microscopy (SEM) observations revealed a 3D flower-like structure for the material extracted from the liquid|liquid interface, identified as CoOOH *vide infra* by X-ray diffraction (XRD) and Raman spectroscopy, with a flower size in the range of 1.5–2.5 μm. Each flower was composed of micron-sized 2D nanosheets. As noted, after calcination, CoOOH fully converted to Co_3_O_4_, and it is important to emphasize that the sample morphology remained unchanged by calcination (Figure 2B). Figure 2C shows the XRD profile of the material extracted from the liquid|liquid interface. The broad diffraction peaks are attributed to the β-CoOOH structure with a hexagonal structure and poor crystallinity, and the peaks are consistent with the standard JCPDS file No. 72-2280.^45^ After calcination (Figure 2D), the spectrum appears significantly different from that shown in Figure 2C and the diffraction peaks become much narrower with significantly improved crystallinity. The diffraction peaks observed at 19° (111), 31.3° (220), 36.9° (311), 38.4° (222), 44.8° (400), 55.7° (422), 59.5° (511), and 65.4° (440) are attributed to the cubic spinel-type structure of Co_3_O_4_ according to standard JCPDS card no: 43-1003.^46^ We note that while β-CoOOH is a layered material, the product after calcination, cubic Co_3_O_4_, is not layered. No other impurity peaks were detected, confirming that β-CoOOH is fully transformed into Co_3_O_4_ after calcination. These results demonstrate that Co_3_O_4_ sheets with high purity are obtained using the present synthesis strategy.

The Raman spectrum of the interfacially grown CoOOH before calcination contains two characteristic peaks, the intense vibration at 503 cm^–1^ and a weaker vibration at 636 cm^–1^ that are attributed to the A_1g_ and E_g_ vibrational modes of CoOOH (Figure 2E).^47^ The FTIR spectrum of the CoOOH 2D nanosheets (Figure S2A) displays three distinct peaks at 3440, 1610, and 655 cm^–1^, which correspond to the bond stretching vibrations of the hydrogen–bonded hydroxyl group (−OH), double bond of Co–O, and Co–O_2_ complex in the oxide.^48^ Note that structural changes occur after calcination, and the new Raman spectral features can be ascribed to spinel-type Co_3_O_4_ (Figure 2F). Specifically, the five signature Raman bands are observed at 185 (F_2g_), 470 (E_g_), 510 (F_2g(2)_), 608 (F_2g(1)_), and 676 (A_1g_) cm^–1^ are attributed to the Co_3_O_4_ spinel structure.^49^ A_1g_ Raman mode relates to octahedral sites, and the E_g_ and F_2g_ modes are believed to be influenced by the vibrations of oxygen atoms bonded to Co^2+^ and Co^3+^ ions located in tetrahedral and octahedral sites. These data are consistent with the FTIR spectrum (Figure S2B), which shows two distinct peaks at ∼660 and 549 cm^–1^, due to the stretching vibration modes of metal oxides. These peaks correspond to tetrahedrally coordinated Co^2+^ and octahedrally linked Co^3+^ metal ions, respectively.^50^

**Figure 3:**
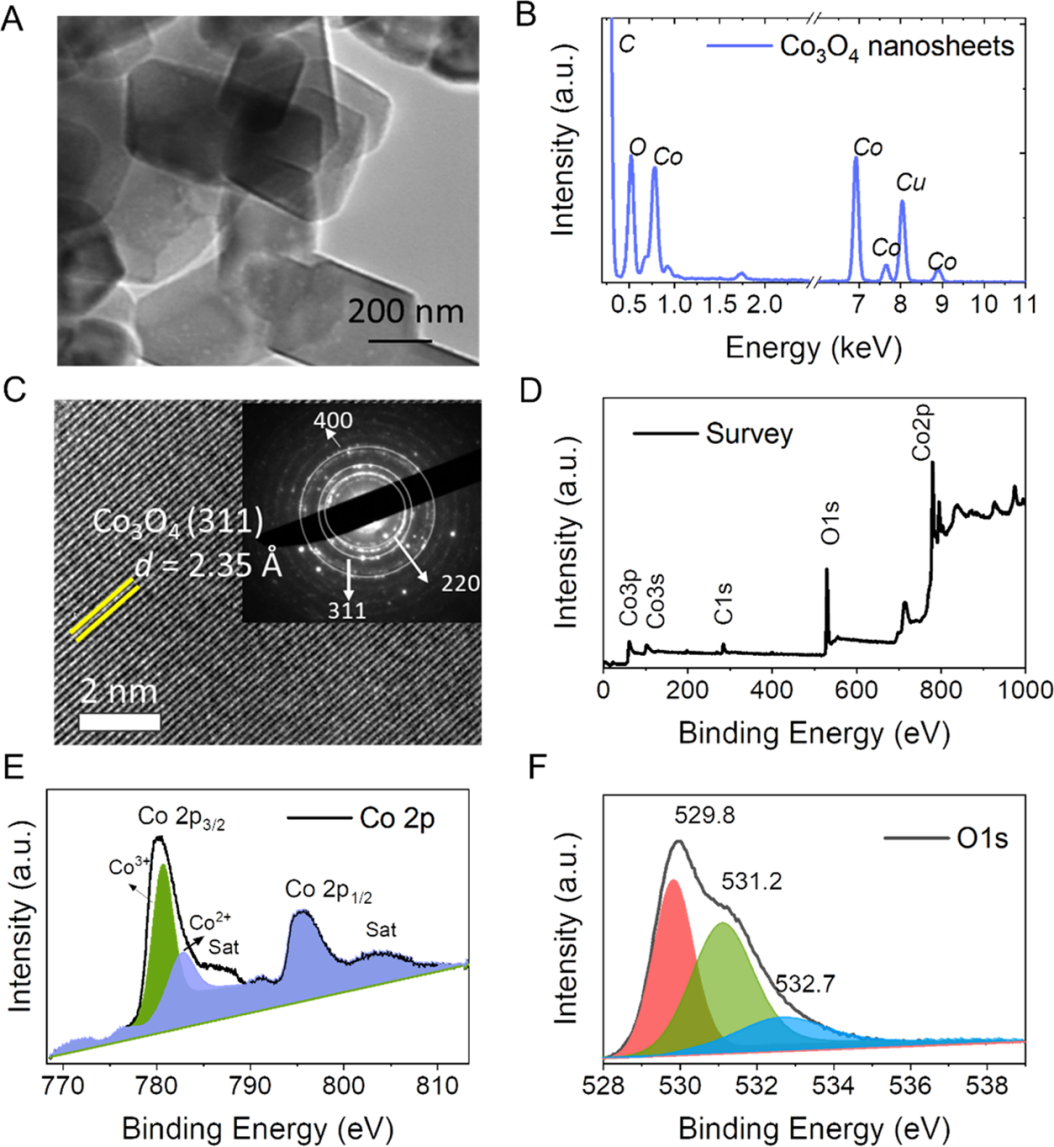
Structural characterization of Co_3_O_4_ 2D nanoplatelets obtained after liquid processing of the Co_3_O_4_ 3D flower-like nanostructures by sonication. (A) Low-magnification bright-field TEM image of Co_3_O_4_ 2D nanoplatelets. (B) EDX spectra on individual 2D nanoplatelets confirmed the existence of Co and O elements. (C) High-resolution scanning transmission electron microscopy (HR-STEM) image obtained from the 2D-flake of Co_3_O_4_, and atomic planes are identified as (311) hkl planes. Inset: Selected area electron diffraction pattern corresponds to the atomic planes of (400), (220), and (311) rhombohedral crystal lattice of Co_3_O_4_. (D) XPS survey spectra of Co_3_O_4_ 2D nanoplatelets. (E) High-resolution Co 2p spectra and (F) deconvoluted O 1s spectra for Co_3_O_4_ 2D nanoplatelets.

The Co_3_O_4_ flowers can be converted into quasi-2D nanoplatelets by ultrasonication of the Co_3_O_4_ powder in IPA for 1.5 h to produce stable nanoplatelet dispersions. To examine the resultant sheets by TEM, a sample was prepared by drop-casting the ultrasonicated dispersion onto a TEM grid. As shown in Figure 3A, the material consists of 2D nanoplatelets with lengths typically in the range of hundreds of nanometers (Figure 3A). The chemical composition and stoichiometry of the sample were examined by measuring energy-dispersive X-ray spectroscopy (EDX) spectra on a nanoplatelet by nanoplatelet basis. In Figure 3B, there is an example of a spectrum that confirms the presence of cobalt and oxygen. The copper and carbon signals originated from the TEM grid. The elemental stoichiometries were extracted from 23 individual spectra. The resultant atomic ratio of cobalt/oxygen was found to be 3:4. This ratio is in line with expectations and is plotted as a histogram in Figure S3. The absence of any other elements suggests that the Co_3_O_4_ 2D nanoplatelets are highly pure. The HR-TEM imaging in Figure 3C depicts clear fringes with 0.235 nm spacing that corresponds to the spinel structure of the Co_3_O_4_ nanoplatelet.^51^ The selected area electron diffraction (SAED) pattern, which can be seen in the inset of Figure 3C, was taken from a region of this platelet. The SAED pattern shows characteristic spot-ring-type diffraction spots that confirm the polycrystalline properties of the Co_3_O_4_ 2D nanoplatelets. The electron microscopy investigation thus confirms, in agreement with the XRD and Raman results, the formation of a polycrystalline Co_3_O_4_ spinel phase.

By performing X-ray photoelectron spectroscopy (XPS) analysis on filtered films of 2D nanoplatelets, we are able to gain a deeper understanding of the chemical composition and electronic structure of the Co_3_O_4_ spinel phase. The XPS survey spectra (Figure 3D) reveal the presence of cobalt and oxygen. The Co 2p XPS spectrum of the 2D nanoplatelets displays two distinct features, one at 795.2 eV and the other at 780.1 eV, which correspond to the spin–orbit peaks of 2p_1/2_ and 2p_3/2_ for Co_3_O_4_, respectively. The Co 2p_3/2_ band with high intensity is curve-fitted into two Co spaces at 782.9 and 780.7 eV, which can be identified as Co^2+^ and Co^3+^ oxidation states. This provides evidence for the existence of both Co^2+^ and Co^3+^ oxidation states (Figure 3E).^49,52^ The Co 2p_3/2_ and Co 2p_1/2_ peaks are separated by a spin–orbit splitting of approximately 15.2 eV. This indicates that Co(II) and Co(III) species coexist. The presence of low-intensity satellite peaks at 783.3 and 804 eV, along with two main Co 2p_3/2_ and Co 2p_1/2_ bands, suggests that the cobalt ions are arranged in a spinel-type lattice structure.^49^ The deconvolution of the O 1s spectra (Figure 3F) reveals three peaks, the intense peak at 529.8 eV for the main lattice oxygen and other peaks at 531.2 and 532.7 eV, which corresponds to the number of lower oxygen coordination sites. The observed peaks and positions are consistent with nanostructured Co_3_O_4_ is a mixed valence compound, specifically Co^(II)^Co_2_^(III)^O_4_, as expected.^49,52^

### Application of Co_3_O_4_ 2D Nanoplatelets in Anodes of Li- and Na-Ion Batteries

#### Preparation of Co_3_O_4_/SWCNT Nanocomposite Anodes

To test the utility of soft-interface-synthesized 2D platelets of Co_3_O_4_ produced in this work, we used them to fabricate and subsequently test both Li- and Na-ion battery electrodes. Such experiments are useful for three reasons. First, they allow us to demonstrate the utility of these materials in an important application area. Second, a comparison of the performance of these materials in both Li and Na storing electrodes allows us to make quantitative comparisons between ion transport and storage for the two ion types. Third, characterizing the electrochemical storage performance of 2D platelets in battery electrodes allows us to test the quality of the materials. As the theoretical capacity is determined by its elemental composition alone, achieving near-theoretical Li/Na-ion storage capacities from these materials would indicate their elemental purity.

As summarized in Table S1, most reports on Co_3_O_4_ show capacities well below the theoretical value and often display capacity degradation with cycling. This is generally attributed to a range of factors including the geometry of the active particles, the low electronic conductivity of Co_3_O_4_, the large volume expansion accompanying the conversion reaction, and the pulverization of the electrode during repeated cycling. Recently, incorporating Li-storing active materials in the form of 2D or quasi-2D particles into electrodes has been shown to achieve extremely high capacities. This is particularly evident when single-walled carbon nanotubes (SWCNTs) are used in place of both the conductive additive and polymeric binder.^36^ Such an electrode architecture yields high out-of-plane electrode conductivity, facilitating charge delivery and allowing the capacity and rate capability to be maximized.^32,38,53^ In addition, the mechanically tough SWCNT network allows the electrode to cope with both volumetric and morphological changes.^26,38^

**Figure 4:**
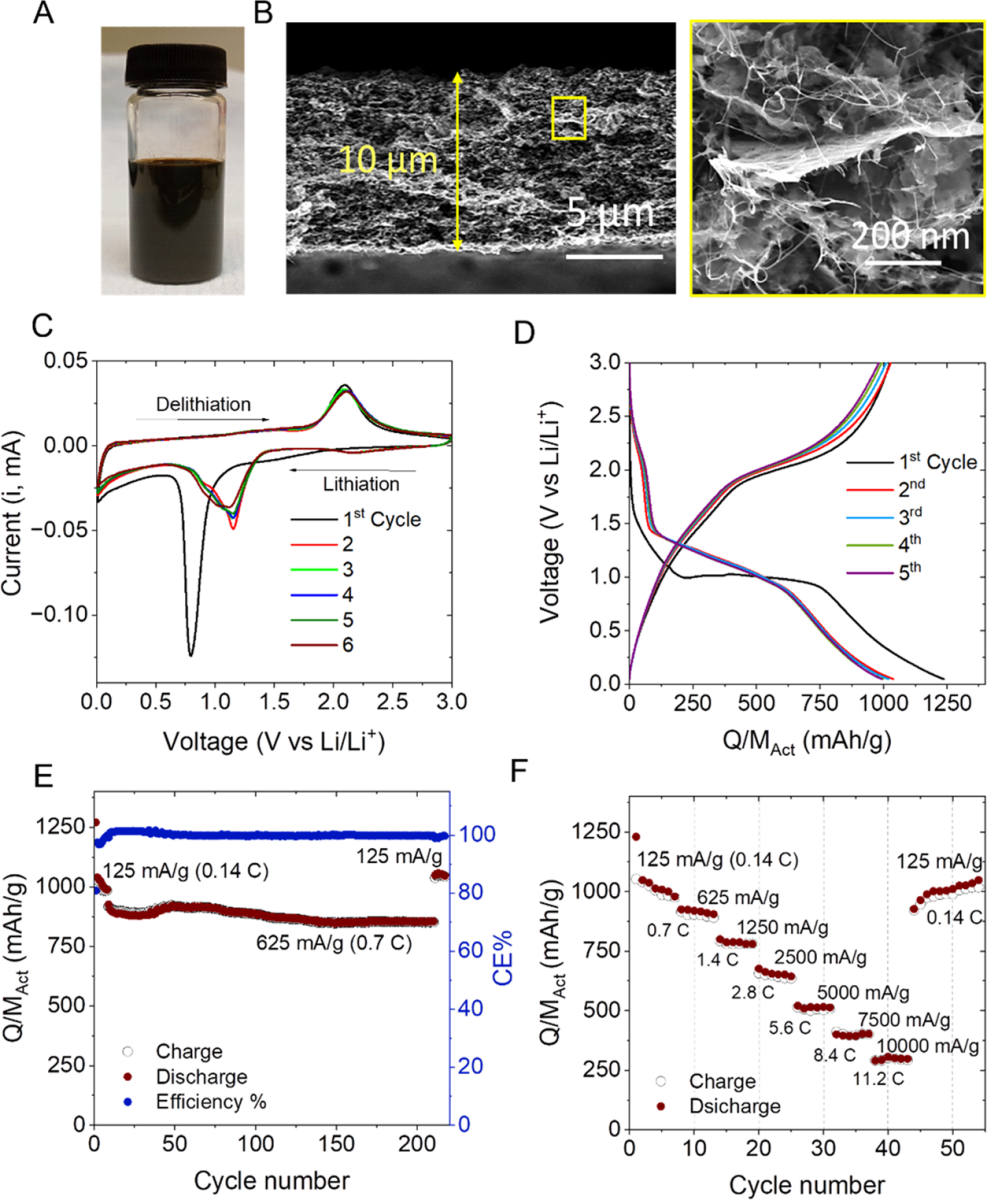
Electrochemical performance of Co_3_O_4_ 2D nanoplatelet/SWCNT composites with *M*_f_^CNTs^ = 20%, area = 0.178 cm^2^, and M_T_/A = 1 mg cm^–2^ as lithium-ion battery anodes. (A) Photograph of the Co_3_O_4_ 2D nanoplatelet dispersion after liquid exfoliation. (B) SEM cross section image of the Co_3_O_4_/SWCNT nanocomposite film at two magnifications. The approximate electrode thickness of the Co_3_O_4_/SWCNT nanocomposite film was 10 μm. (C) Cyclic voltammograms recorded for the first 6 cycles at a sweep rate of 0.1 mV/s for the Co_3_O_4_/SWCNT nanocomposite anode. (D) Charge–discharge voltage plateaus collected at the first 5 cycles for the Co_3_O_4_/SWCNT nanocomposite anode. (E) Charge–discharge cycling capacity for the Co_3_O_4_/SWCNT composite anodes cycled at 125 mA/g for the initial 6 cycles, followed by 200 cycles at 625 mA/g. (F) Rate performance of the Co_3_O_4_/SWCNT nanocomposite anode at various specific currents as a function of cycle number.

Co_3_O_4_/SWCNT nanocomposite anodes were created by mixing Co_3_O_4_ 2D nanoplatelets (see the Experimental Methods section) with SWCNT dispersions (20 wt %) and vacuum filtering the mixture onto Celgard 2320 membranes. This process produced composite films with an areal mass loading of approximately 1 mg/cm^2^ (Figure S4A). The films were then cut into the necessary dimensions for electrochemical testing with an area of 0.178 cm^2^. From the cross-sectional SEM images of such films (Figure 4B), the electrodes were close to 10 μm thick and uniformly mixed with well-dispersed SWCNT networks. This thickness, combined with the mass loading, implies an electrode density of ∼1 g/cm^3^, which is much less than the Co_3_O_4_ density (6.1 g/cm^3^) and therefore implies that the porosity of these films is >80%. We observed that the electrodes used for electrochemical testing were extremely porous, significantly higher than the values of ca. 50–60% found for previous nanosheet/nanotube composites.^54^

#### LIB Performance of Co_3_O_4_/SWCNT Nanocomposite Anodes

The Co_3_O_4_/SWCNTs nanocomposite anodes were first examined using cyclic voltammetry (CV) in the potential range 0.05–3.0 V to determine their electrochemical properties, at a sweep rate of 0.1 mV/s in a half-cell assembled with a fresh Li disk as a reference. The initial 6 cycles of the representative CV curves of the electrodes are presented in Figure 4C and are consistent with the following electrochemical conversion reactions^55,56^
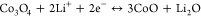
1a
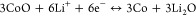
1b

The overall reaction is therefore Co_3_O_4_ + 8Li^+^ + 8*e*^–^ ↔ 4Li_2_O + 3Co. In the first discharge cycle, a broad cathodic peak was observed at ∼0.8 V that disappears in subsequent cycles and is most likely due to the extra Li-ion adsorption/desorption during the formation of a solid electrolyte interface (SEI) on the Co_3_O_4_/SWCNT electrode surface. Subsequently, this main reduction peak is shifted to ∼1.15 V, which can be assigned to the lithiation of Co_3_O_4_, *i.e*., electrochemical reduction of Co_3_O_4_ to metallic cobalt with the accompanying formation of an amorphous Li_2_O matrix. In the anodic processes, the anodic peak at 2.1 V is ascribed to the delithiation reaction of Co_3_O_4_, *i.e*., electrochemical oxidation of Co to reform Co_3_O_4_. On the other hand, this oxidation peak at ∼2.1 V remained virtually identical during the first 6 cycles, suggesting that the SEI formed on the Co_3_O_4_/SWCNTs electrode surface in the first cycle is very stable. Beyond the first cycle, the overlapping CV curves in subsequent cycles suggest good electrochemical conversion reaction reversibility and stability of the electrochemical conversion reaction(s) of Co_3_O_4_.

Galvanostatic charge–discharge (GCD) measurements were performed to evaluate the Li-storage capacity of the Co_3_O_4_/SWCNT nanocomposite anodes. Unless specified otherwise, the discharge capacity and current density were determined by considering the weight of the active material (*M*_Act_) and are expressed as *Q*/*M*_Act_. Figure 4D displays the voltage profiles associated with the first 5 standard activation cycles at low specific current (*I*/*M*_Act_ = 125 mA/g). After this, 200 cycles were carried out at a higher specific current (*I*/*M*_Act_ = 625 mA/g). The purpose of the activation cycles is to allow the SEI layer to form on the electrode surface. For the first cycle, discharge profiles show a plateau at about 1.0 V (vs Li/Li^+^), which shifts to 1.28 V for the following cycles and corresponds to the electrochemical conversion reaction from Co_3_O_4_ through an intermediate phase of CoO and then to metallic Co, respectively. The voltage plateau moves up and becomes higher than that of the first cycle, and the voltage plateau becomes steep in the subsequent discharge curves, which was attributed to the multistep reactions and the occurrence of some irreversible transformation and structural change in the first cycle.^57^ This is a common phenomenon in the reported results of the TMO electrodes for LIBs. In the charging curves, the reactions are reversed. It is noteworthy that the first discharge voltage plateaus are different at the CV and GCD curves, possibly because of the difference in the operational conditions (i.e., current density and batch to batch cells testing); the processes associated with the first discharge cycle such as irreversible electrolyte decomposition as well as structural pulverization and entrapment of excess lithium in the active material may not be identical in the first CV and GCD test. We have repeatedly found this lithiation voltage difference in the first discharge cycle for many Co_3_O_4_-based nanostructures as anodes for LiBs.^58,59^ Apart from the first cycle, charge–discharge plateaus are generally consistent with the redox peak potentials observed in the CV curves shown in Figure 4C (see Figure S4B for differential voltage plateaus). In the subsequent cycles, charge–discharge curves are virtually identical and tend to be stable, indicating the stability of the nanostructures as anode materials. We note that, similar to Fe_2_O_3_, the Co_3_O_4_ operating voltage is still quite high, which hinders its potential use in real anodes.

The first cycle in Figure 4E of the GCD curves at 125 mA/g (at 0.14 C, where 1 C = 890 mAh/g) shows a very high initial discharge capacity of 1271 mAh/g, with 1027 mAh/g recovered after the first full charge with a Coulombic efficiency (CE) of 80%. These values are much larger than the theoretical capacity of bulk Co_3_O_4_ (∼890 mAh/g). Such high-capacity values and the initial irreversible capacity loss during the first cycle are mainly due to the inevitable formation of the SEI layer and the irreversible degradation of the electrolyte. During the next 5 activation cycles, the discharge capacity of the electrode gradually decreased from 1040 to 1009 mAh/g with better electrochemical performance, while the Coulombic efficiency rose to 99%, indicating stable SEI formation. When the current was increased to 625 mA/g (at 0.7 C), good cycling performance was observed, and the electrodes showed stability at ca. 880–850 mAh/g with a CE of >99% for 200 cycles of operation. On the 211th cycle, the current was reduced to 125 mA/g again. The composite electrode regained its initial capacity of 1049 mAh/g and achieved a CE of over 99%. This shows the effective reversibility of the electrochemical lithiation/delithiation reactions and excellent cyclic charge–discharge performance with high CE. It is important to assess the capacity contribution from the CNTs, which we do via literature data, as shown in Figure S5. This shows the specific capacity of the SWCNTs for Li is ∼400 mAh/g and for Na is ∼90 mAh/g at 100 mA/g. Then, the maximum contribution of SWCNTs in our electrodes (in both cases, 20 wt % SWNTs used) is 80 mAh/g for Li and 22 mAh/g for Na. These are relatively small contributions to the overall electrode capacity (over 1000 mAh/g) in line with previous work.^37,60^

Rate capability was evaluated at various current densities between 125 and 10 000 mA/g (0.14 and 11.2 C) as shown in Figure 4F. It can be found that the discharge and charge capacities remain stable and show the expected reduction in capacity with increasing charge–discharge current. However, especially at low currents, the constant current steps show a small capacity fade, and in subsequent cycles, these data show reasonably good stability and an almost complete recovery of the reversible capacity when the current density is decreased from 11.2 to 0.14 C (*i.e*., cycles 44 to 54). We will analyze the rate data in more detail *vide infra*.

#### SIB Performance of Co_3_O_4_/SWCNT Nanocomposite Anodes

**Figure 5:**
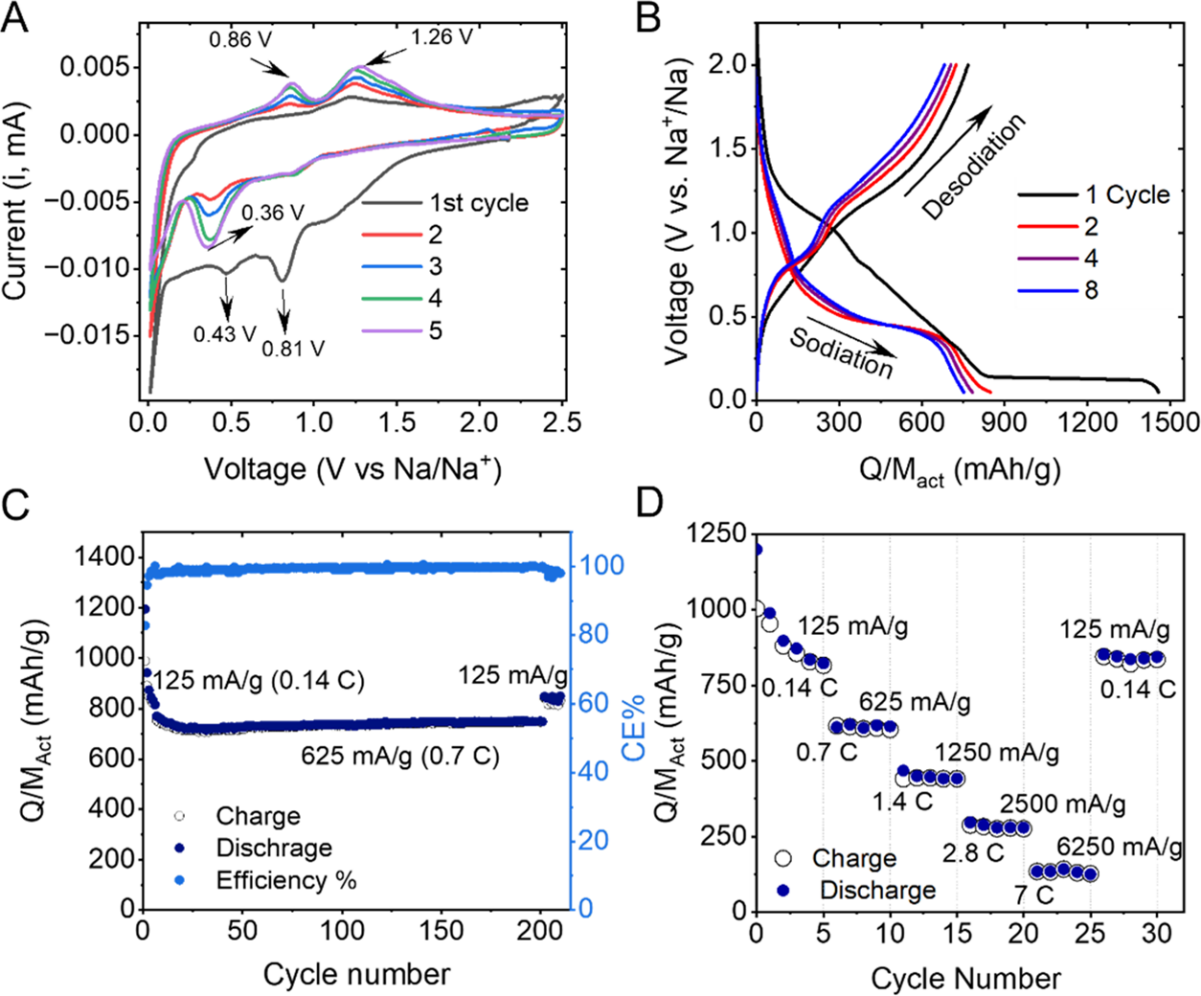
Electrochemical performance of Co_3_O_4_ 2D nanoplatelet/SWCNT composites with *M*_f_^CNTs^ = 20%, area = 0.178 cm^2^, and *M*_T_/*A* = 1 mg cm^–2^ as sodium-ion battery anodes. (A) Cyclic voltammograms recorded for the first 5 cycles at a sweep rate of 0.1 mV/s in the potential range of 0.05–2.5 V for the Co_3_O_4_/SWCNT nanocomposite anode. (B) Charge–discharge plateaus collected for the first 5 cycles for the Co_3_O_4_/SWCNT nanocomposite anode. (C) Charge–discharge cycling performance for the Co_3_O_4_/SWCNT composite anode cycled at 125 mA/g for the initial 5 cycles and 625 mA/g for the subsequent 200 cycles. (D) Rate performance of the Co_3_O_4_/SWCNT nanocomposite anode at various specific currents as a function of cycle number.

Subsequently, the Co_3_O_4_ 2D nanoplatelet/SWCNT composites were characterized as SIB anodes, mirroring the experiments performed above for LIBs. The electrochemical Na-storing properties of the Co_3_O_4_/SWCNTs composite anodes were first examined using CV, in the potential range of 0.05–2.5 V (sweep rate of 0.1 mV/s) in a half-cell assembled with Na foil (Figures 5 and S6). The initial 5 cycles of the representative CV curves of the electrodes are presented in Figure 5A. In the first cycle, two broad cathodic peaks observed at 0.81 and 0.43 V are due to SEI layer formation, the electrochemical formation of Na_2_O, and the reduction of Co_3_O_4_ to metallic Co. These values are less positive than those observed in the CV curves for LIBs. As demonstrated in the literature, the conventional charge–discharge potentials for common hosts are lower for Na as compared to Li. This leads to slower reaction kinetics in SIBs due to the larger ionic radius of Na^+^, which will result in slower diffusion.^56,61^ In the anodic region, peaks are observed around 0.86 and 1.26 V that correspond to the oxidation of Co to Co_3_O_4_ and decomposition of Na_2_O.^56^ The overall electrochemical conversion reaction of Co_3_O_4_ with Na is the same as that with Li and can be expressed as the following two steps^56^
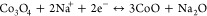
2a
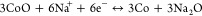
2b

The SEI layer is formed during the first cycle only, as from the second cycle the peak at 0.81 V disappeared and the reduction peak at 0.43 V shifted to 0.38 V in the cathodic region. We note that the lower operating potential of Co_3_O_4_-based electrodes for Na compared to Li could yield advantages in terms of full-cell energy density for SIBs compared to LIBs.

To further evaluate the Na-storage capacity of these nanocomposite electrodes, we performed GCD measurements with a potential range of 0.05 to 2.5 V. The charge and discharge profiles at 125 mA/g in Figure 5B are generally consistent with the CV profiles shown in Figure 5A. Beyond the first discharge cycle, the charge and discharge curves are significantly overlapping, which indicates the stability of the nanostructures as an anode. The cycle performance of the Co_3_O_4_/SWCNTs nanocomposite electrodes was investigated at a current density of 125 mA/g (at 0.14 C) for the initial 6 cycles and at 625 mA/g (at 0.7 C) for the subsequent 200 cycles, as shown in Figure 5C. The first cycle shows very high initial discharge and charge capacities of approximately 1249 and 990 mAh/g, respectively, resulting in an initial CE of ∼79.3%. As for the LIBs, this initial irreversible capacity loss of the nanocomposite electrode is associated with the inevitable decomposition of electrolyte and formation of SEI.^55^ During the next five activation cycles, the reversible discharge capacity gradually stabilized at 815 mAh/g with a Coulombic efficiency of 98.7%. However, on the seventh cycle, when the current was increased to 625 mA/g, there was a significant reduction in capacity. Overall, the nanocomposite electrode exhibits excellent cycling performance, maintaining a stable capacity of 735 mAh/g.

The capacity and CE (∼99%) remain quite stable when the Co_3_O_4_/SWCNT nanocomposite anode was cycled at a high current density (at 625 mA/g) over 200 cycles (Figure 5C). By the end of the cycling process, the electrodes fully recovered their original low-rate capacity of 830 mAh/g, indicating good reversibility of the electrochemical performance. The rate performance measurements were evaluated at various current densities between 125 and 6250 mA/g (at 0.14C and 7C) as shown in Figure 5D. The composite electrodes display the expected reduction in capacity with increasing charge–discharge current. However, as with Li, while the constant current steps at low currents show a minor capacity fade, in subsequent cycling, these data show reasonably good stability and an almost complete recovery to the initial low-rate capacity after the rate performance test (*i.e*., cycles 26 to 30).

In order to prove the structural stability of the Co_3_O_4_/SWCNT composite electrode, the SEM cross-sectional images were examined after 200 cycles for both Li- and Na-ion anodes (Figure S7). After cycling, the electrodes appear smoother with no visible 2D platelets, indicating a morphological transformation to a more uniform, amorphous structure.

#### Quantitative rate performance analysis

**Figure 6:**
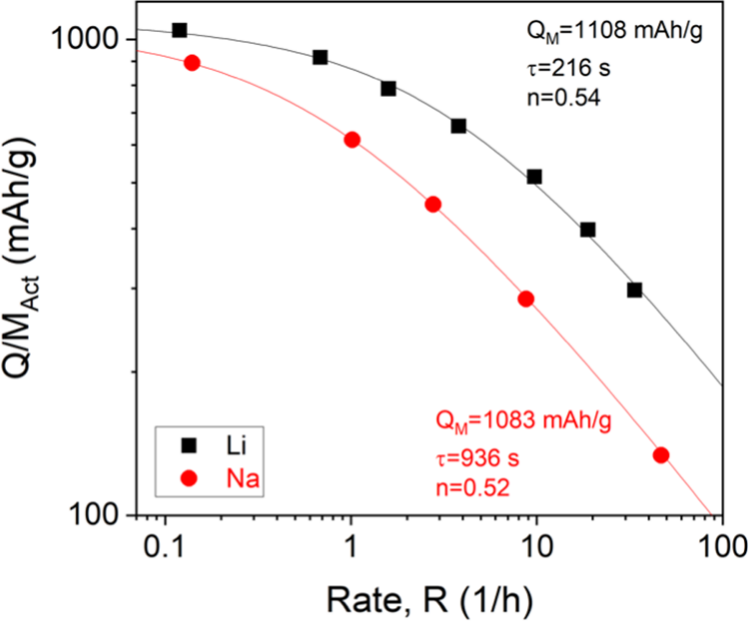
Quantitative rate analysis for Co_3_O_4_ 2D nanoplatelet/SWCNT composites with *M*_f_^CNTs^ = 20%, area = 0.178 cm^2^, and *M*_T_/*A* = 1 mg cm^–2^ as both lithium- and sodium-ion battery anodes. The capacity versus rate curves were fit to eq 5 with the fit parameters shown in the figure.

We have previously shown that much information about the ultimate capacity of electrodes and the factors limiting their rate performance can be obtained by quantitative analysis of capacity versus rate data.^62−65^ To achieve this, the measured specific capacity (*Q*/*M*_Act_) for Li- and Na-storing Co_3_O_4_/SWCNT nanocomposite electrodes is plotted versus the rate in Figure 6. We represent charge–discharge rate using the parameter, *R*, which is defined as *R = I*/*Q*, *i.e*., the ratio of the specific current to the specific capacity.^62,63,65−67^ The advantage of this approach is that 1/*R* is a measure of the actual charge–discharge time of the electrode at a constant current. This data shows that the measured capacity falls off with rate (*R*) as is generally observed.^64,65^

To analyze this data, we use a semiempirical fitting equation proposed recently by us^65^
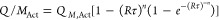
3to yield three fit parameters: *Q*_M,Act_, the low-rate capacity, which can be compared to the theoretical capacity (normalized to active mass); τ, the characteristic charge–discharge time; and *n*, a parameter whose value indicates whether diffusive (*n* = 0.5) or electrical (*n* = 1) limitations dominate rate performance.^62−66^ As shown in Figure 6, this equation fits both data sets extremely well. The obtained fit values were *Q*_M,Act_ = 1108 mAh g^–1^, *n* = 0.54, and τ = 216 s for the Li-storing electrodes and *Q*_M,Act_ = 1083 mAh g^–1^, *n* = 0.52, and τ = 936 s for the Na-storing electrodes.

The *Q*_M,Act_ values represent the maximum achievable capacity and, in both cases, are very close to the theoretical capacity (980 mAh/g). This indicates that the combination of 2D nanoplatelets of Co_3_O_4_ with the use of SWCNTs as both binder and conductive additive allows full utilization of the active material for the storage of both ion types. This is consistent with a number of previous studies on various 2D material-based electrodes.^26,32,37,38,68^ In both cases, the *n*-values are very close to 0.5, indicating that both electrode types are diffusion-limited, at least for this electrode thickness (∼10 μm).

The time constants, τ, represent the minimum charge–discharge time for the electrode and can be used as a metric for rate performance, with higher values of τ indicating poorer rate performance. These values are clearly different, with the Li-storing electrodes displaying a τ-value roughly four times lower than their Na-storing counterparts. However, this does not necessarily imply a proportionate difference in rate performance as the systems have slightly different experimental parameters, *i.e*., different separator thicknesses (see the Experimental Methods section and the Supporting Information). Therefore, a quantitative rate analysis is required to extract the intrinsic differences between the systems.

The characteristic time associated with charge–discharge, τ, can be used to obtain significant insights into the factors limiting the rate performance. Recently, we have shown that τ has a number of contributions from both capacitive/resistive and diffusive terms and can be described quantitatively by an equation^62^ which links it to various physical parameters associated with the cell. Here, this equation is presented in a way that highlights the various capacitive (whose sum is τ_C_) and diffusive (whose sum is τ_D_) terms
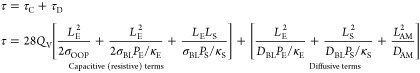
4where *Q*_V_ is the electrode low-rate volumetric capacity, *L*_E_ is the electrode thickness, σ_OOP_ is the electrode out-of-plane electronic conductivity, σ_BL_ is the bulk electrolyte ionic conductivity, *D*_BL_ is the ion diffusion coefficient in the bulk electrolyte, and *L*_S_ is the thickness of the separator. *P*_E_ and κ_E_ are the porosity and tortuosity of the electrode, while *P*_S_ and κ_S_ are the equivalent values for the separator, respectively. *L*_AM_ is the solid-state diffusion length (related to active particle size), and *D*_AM_ is the Li/Na-ion diffusion coefficient within the active particles (*D*_AM_ is an effective value, averaged over all states of charge).

This equation is particularly useful as many of the parameters therein are known (*e.g*., *D*_BL_) and can be measured (*e.g*., *L*_E_) or estimated (*e.g*., κ_E_). As shown in Table S2, we have tabulated known values or good estimates of all of these parameters except the solid-state diffusion coefficients (*D*_AM_) for Li and Na ions in Co_3_O_4_. By combination of these values with eq 4, the values of *D*_AM_ that are consistent with the measured values of τ for the Li- and Na-storing Co_3_O_4_ electrodes may be calculated. This analysis implies values of *D*_AM_ of 1.5 × 10^–17^ m^2^/s and 6.5 × 10^–18^ m^2^/s for Li and Na, respectively. These values yield τ-values of 221 and 945 s for Li and Na, respectively, close to measured values of 216 and 936 s (see Table S2).^69^

Using the values of *D*_AM_ found above, combined with the parameters given in Table S2, the contributions to the overall time constant were calculated from both capacitive (resistive) and diffusive contributions, τ_C_ and τ_D_, as 44 and 177 s for Li and 106 and 839 s for Na, respectively. The higher values of τ_D_ imply both systems to be predominately diffusion-limited, in agreement with the *n*-values. In fact, these values of τ_C_ and τ_D_ can be used to estimate *n*. If τ_C_ ≫ τ_D_, a value of *n* = 1 is expected, while if τ_C_ ≪ τ_D_, a value of *n* = 0.5 is expected. Thus, we propose that *n* can be approximated by^62^
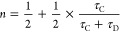
5

Combining this equation with the calculated values of τ_C_ and τ_D_ yields *n*-values of 0.59 and 0.56 for Li and Na, respectively, that are very close to the measured values of 0.54 and 0.52. This further supports our analysis and confirmed our cells to be diffusion-limited.

Having confirmed the diffusion-limited nature of these cells, it is worth considering what form of diffusion is limiting. The three terms in the second square bracket in eq 4 represent time scales associated with (from left to right): diffusion within the pores of the electrode; diffusion within the pores of the separator; and solid-state diffusion within the active material. For electrodes of this thickness, we can work out values of each term to be 0.6, 10, and 167 s, respectively, for the Li-storing electrodes. This makes it clear that the Co_3_O_4_/SWCNT nanocomposite electrodes are completely limited by solid-state diffusion when used as the anode in the LIB. However, it is worth noting that because time for diffusion in the pores of the electrode scales as *L*_E_^2^, the first diffusive time scale will increase rapidly as electrode thickness is increased. Nevertheless, even increasing electrode thickness to 100 μm should not give in-pore diffusive time scales greater than ∼60 s. This means that any practical electrode thickness fabricated by using this material will still result in rate performance being limited by solid-state diffusion.

The situation is slightly different for the Na-storing electrodes, which used much thicker separators (*L*_S_ = 300 μm). For these electrodes, the time scales associated with each type of diffusion were 6 s (electrode pores), 454 s (separator pores), and 384 s (solid state). Here the diffusion time within the separator pores is high, simply due to the large separator thickness. Aside from this contribution, it is clear that solid-state diffusion is dominant. In addition, the solid-state diffusion time is significantly longer than that quoted above for the Li-storing electrodes. This difference is due to the lower solid-state diffusion coefficient associated with Na ions in Co_3_O_4_. We note that if the electrode thickness were increased to the more technologically relevant value of *L*_E_ = 100 μm, this would increase the in-pore liquid diffusion time from 6 to 600 s and the total value of τ from 936 to ∼2500 s. While this is a significant increase, that is an expected effect of increasing electrode thickness and is partially compensated by the accompanying increase in areal capacity.^67^ Under these circumstances, the solid-state diffusion time remains the same but would only be ∼15% of the overall τ-value and so no longer dominant.

## Conclusions

In conclusion, we demonstrated the successful synthesis of Co_3_O_4_ 2D platelets by a three-step route. First, we produced CoOOH in the form of nanoflowers using an electrochemically polarized liquid|liquid interface. Then, calcination of the CoOOH powder recovered from the liquid|liquid interface produced Co_3_O_4_ powder with the same morphological nanostructure as the CoOOH. The structure and stoichiometry of these nanoflowers are consistent with that of the spinel structure of Co_3_O_4_, which is confirmed by XRD, XPS, Raman, SEM, and TEM-EDX analyses. Ultrasonication was used to break up these nanoflowers to produce 2D nanoplatelet dispersions in IPA. HR-TEM characterization confirmed that the nanoplatelets were 2D and identical to the 3D nanoflowers in all aspects, except for their shape. Finally, these Co_3_O_4_ 2D nanoplatelets were characterized as both Li-ion and Na-ion battery anodes by combining them with carbon nanotubes. We observed excellent performance of these anodes for both Li and Na storage with low-rate capacities in excess of 1000 mAh/g in each case. The detailed quantitative rate analysis reveals that Li-ion-storing anodes charge roughly five times faster than Na-ion-storing anodes.

We believe this work is novel for a number of reasons. First, we believe this is the first time 3D flower-like Co_3_O_4_ structures have been produced at a polarized liquid/liquid interface. In addition, this is the first paper reporting the production of 2D nanoplatelets of Co_3_O_4_ by the sonication-induced fragmentation of such flowers. Access to suspensions of these platelets allows us to prepare nanoplatelet/nanotube composites which can be used as battery electrodes without any further binder or conductive additive. The resultant electrodes have impressive low-rate capacities, which are close to or beyond the state of the art for Li-ion or Na-ion Co_3_O_4_ electrodes (Figure S8). Finally, our novel quantitative rate analysis has clearly shown the rate-limiting step to be associated with solid-state diffusion and confirmed the diffusion coefficient of Na ions in Co_3_O_4_ to be considerably slower than that of Li ions. Although the working voltage (∼1 V) in the Li cells is probably too high for real applications, the lower voltage of ∼0.5 V in the sodium cells may be more practical.

## Supporting Information Available

Synthesis of Co_3_O_4_ at the interface for the polarization of ITIES (S1); FTIR spectra of CoOOH and Co_3_O_4_ nanosheets (S2); statistical histogram of O and Co elemental from EDX analysis (S3); Co_3_O_4_/SWCNT composite electrode electrochemical performance as LIB anode (S4); Co_3_O_4_/SWCNT composite electrode electrochemical performance as Na ion (S5); post-cycling SEM analysis (S6); literature comparison of the Co_3_O_4_ nanostructures for both Li-ion and Na-ion battery anodes (Table S1); modeling rate performance (S7); Co_3_O_4_/SWCNT composite electrodes parameters (Table S2) (PDF)
