# Supplementary material for: Cobalt Oxide 2D Nanosheets Formed at a Polarized Liquid|Liquid Interface toward High-Performance Li-Ion and Na-Ion Battery Anodes
Source: ACS Appl Mater Interfaces. 2023 Dec 5;15(50):58320–32. doi: 10.1021/acsami.3c11795 (PMC10739576; doi:10.1021/acsami.3c11795)
Supplement: Supplementary file 1 — am3c11795_si_001.pdf [file am3c11795_si_001.pdf]

Supporting Information

Cobalt Oxide 2D-Nanosheets Formed at a Polarized Liquid|Liquid Interface towards High Performance Li-Ion and Na-Ion Battery Anodes

Bharathi Konkena,[1]* Chakrapani Kalapu,[2] Harneet Kaur,[1] Angelika Holzinger,[3] Hugh Geaney,[3] Valeria Nicolosi,[4] Micheál D. Scanlon,[3]* and Jonathan N. Coleman[1]*

[1]School of Physics, CRANN & AMBER Research Centres, Trinity College Dublin, Dublin D2, D02 K8N4, Ireland

[2]Micro Nano Systems Department, Tyndall National Institute, Cork, T12 R5CP, Ireland

[3]The Bernal Institute and Department of Chemical Sciences, University of Limerick, Limerick V94 T9PX, Ireland

[4]School of Chemistry, CRANN & AMBER Research Centres, Trinity College Dublin, Dublin D2, D02 W9K7, Ireland

Email: *colemaj@tcd.ie;micheal.scanlon@ul.ie; konkenab@tcd.ie

S1. Synthesis of Co_3O_4 at the interface for the polarization of ITIES

S2. FTIR spectra of CoOOH and Co_3O_4 nanosheets

S3. Statistical histogram of O and Co elemental from EDX analysis

S4. Co_3O_4/SWCNT composite electrode electrochemical performance as LIB anode

S5. Specific capacity of SWCNTs for Li and Na-ion battery

S6. Co_3O_4/SWCNT composite electrode electrochemical performance as Na-ion

S7. Post cycling SEM analysis

Table S1. The literature comparison of the Co_3O_4 nanostructures for both Li-ion and Na-ion battery anodes

S7. Modelling rate performance; Table S2. Co_3O_4/SWCNT composite electrodes parameters

S8. State of the art figure for LIBs and SIB literature data

S1. Synthesis of Co_3O_4 at the interface for the polarization of ITIES

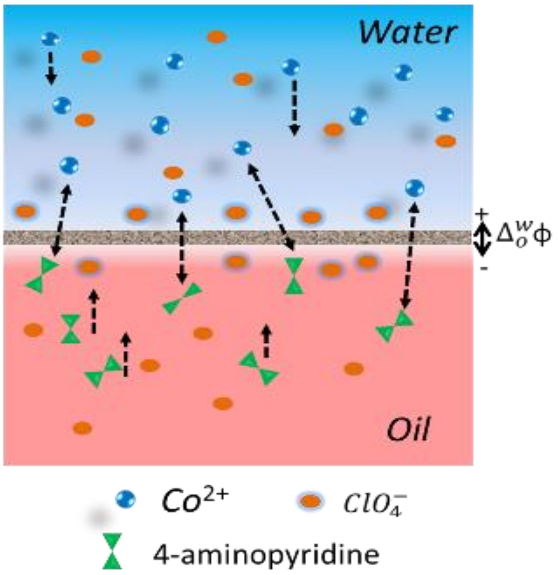

Figure S1. **Schematic representation of the interface for the polarization of ITIES.** The Co_3O_4 microstructures assembled at the water/DCE interface. Interface is electrified/polarized by the common ion, ClO_4^-, that partitioning between the two liquid phases.

ITIES can be polarized either by using a potentiostat externally (electrodes) or chemically by changing the composition or concentration of supporting electrolytes (common ions) in the organic or aqueous phase. Our approach herein is the use of ClO_4^- as a common ion which

induces the specific potential difference across the interface according to the Nernst–Donnan equations.[1] Here, polarisation of the interface is imposed internally, by creating an excess of charges in one phase, compensated by the same excess (of opposite sign) in the other phase, neutralise each other facilitating the reaction between Co^{2+} and 4-amino pyridine.

S2. FTIR spectra of CoOOH and Co_3O_4 nanosheets

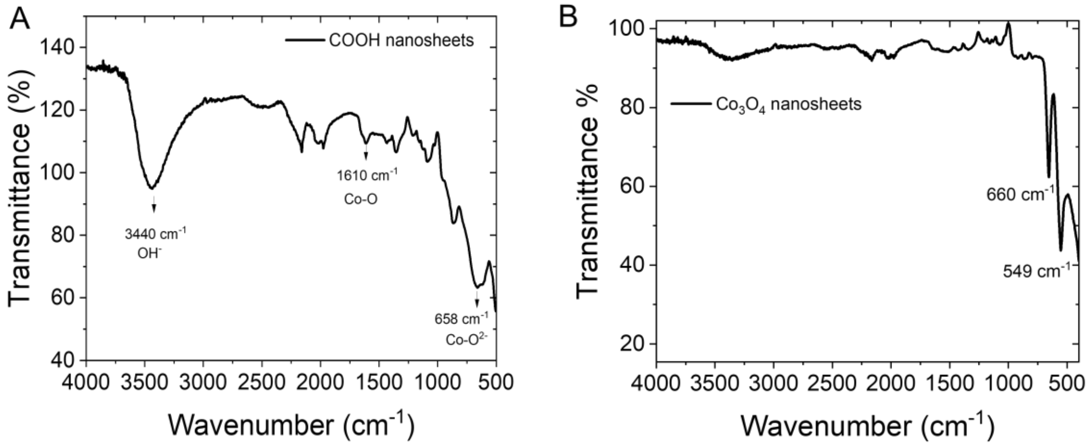

Figure S2. FT-IR spectra of fragmented CoOOH and Co_3O_4 nanosheets. Spectra displaying all the significant expected peaks for CoOOH and Co_3O_4.

S3. Statistical histogram of O and Co elemental from EDX analysis

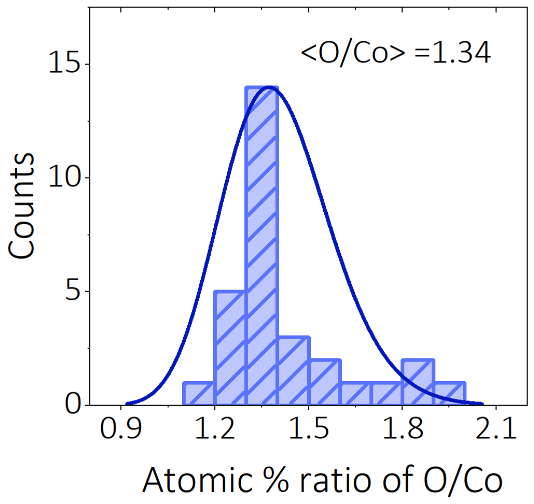

Figure S3. Statistics of O and Co elemental compositions obtained from EDX analysis using TEM over the 20 individual platelets.

S4. Co_3O_4/SWCNT composite electrode electrochemical performance as LIB anode

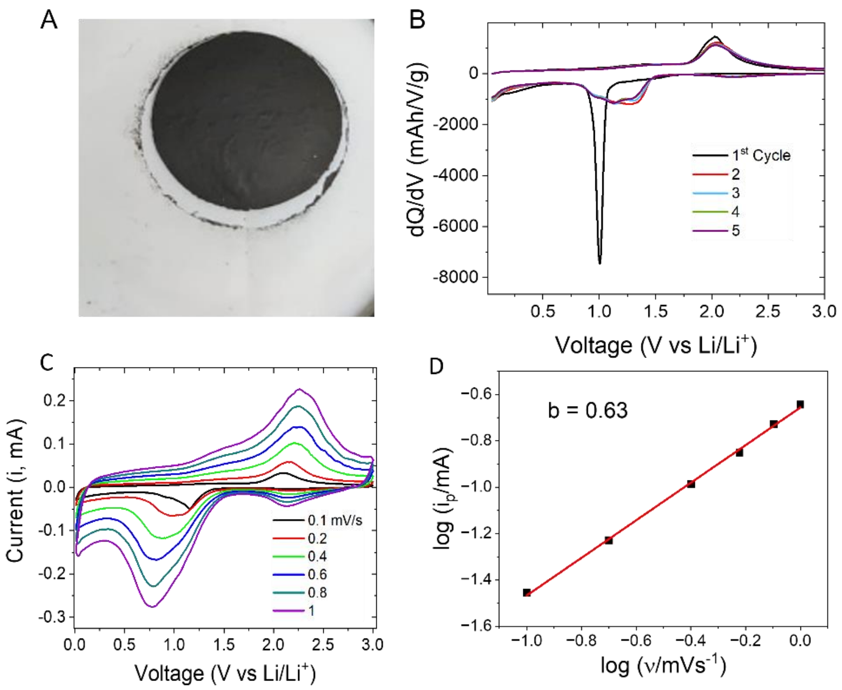

Figure S4. Electrochemical performance of Co_3O_4/SWCNT composite electrodes as Li-ion battery anode. (A) Free-standing vacuum filtered Co_3O_4/SWCNTs composite film used for electrochemical testing. (B) Charge-discharge dQ/dV curves corresponding to the first six cycles at 125 mA/g (i.e., first 5 activation cycles) shown in the Figure 4D for 6 µm thick Co_3O_4/SWCNT composite electrodes (C) Cyclic voltammograms collected at various scan rates from 0.1 to 1 mV s^{-1} for Co_3O_4/SWCNT composite electrodes. (D) Log(i_p) versus log(ν) plot of the cathodic current response (i_p measured at 0.75 V).

S5. Specific capacity of SWCNTs for Li and Na-ion battery

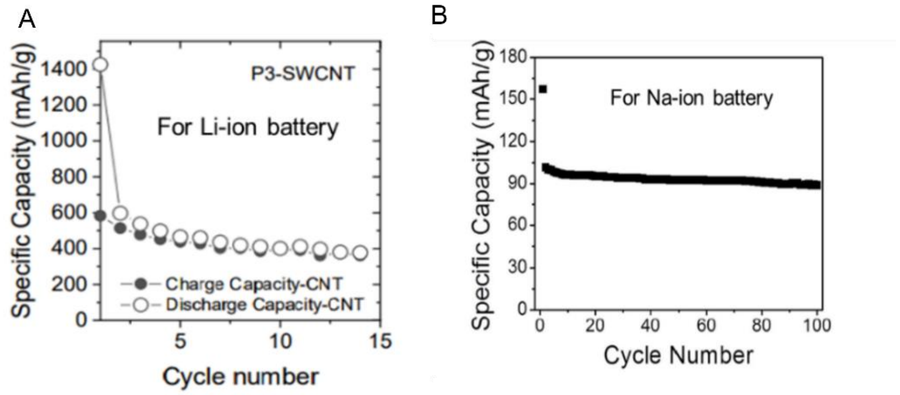

Figure S5. Cycling performance of SWCNTs film alone (A) for Li, and (B) for Na at a current density of 100 mA/g.

S6. Co_3O_4/SWCNT composite electrode electrochemical performance as Na-ion

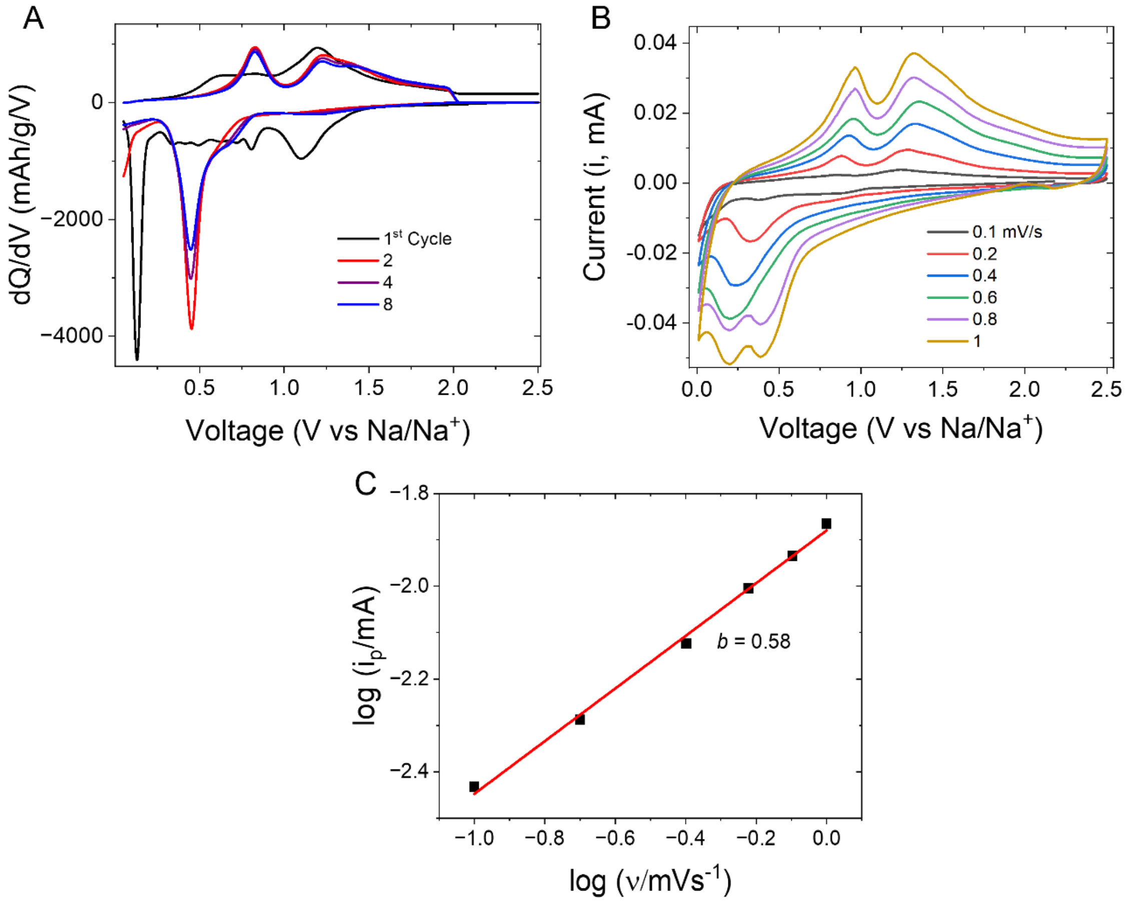

Figure S6. Electrochemical performance of Co_3O_4/SWCNT composite electrodes as Na-ion battery anode. (A) Charge-discharge dQ/dV curves corresponding to the first six cycles at 125 mA/g (i.e., first 5 activation cycles) shown in the Figure 4D for 6 μm thick Co_3O_4/SWCNT composite electrodes (B) Cyclic voltammograms collected at various scan rates from 0.1 to 1 mV s^{-1} for Co_3O_4/SWCNT composite electrodes. (C) Log(i_p) versus log(ν) plot of the cathodic current response (i_p measured at 0.75 V).

S7. Post cycling SEM analysis

SEM cross section images of the Co_3O_4/SWCNT composite electrode were examined before (figure 4B) and after cycling (figure S7). The most obvious difference noticed here is electrode has expanded from 10 mm to 16.2 mm after cycling for Li-ion battery anode while for Na-ion battery anode expanded to 20 mm. The morphology of the Co_3O_4/SWCNT composite electrode appears significantly different to that shown in Figure 4B. This electrode expansion implies that the reduction of density implying an increase in porosity. Post cycling electrodes appears much smoother with no 2D-platelets visible, implying a morphological change to a more uniform, amorphous structure. It is found that the shape of the conversion type materials will not remain same after cycling.[2, 3]

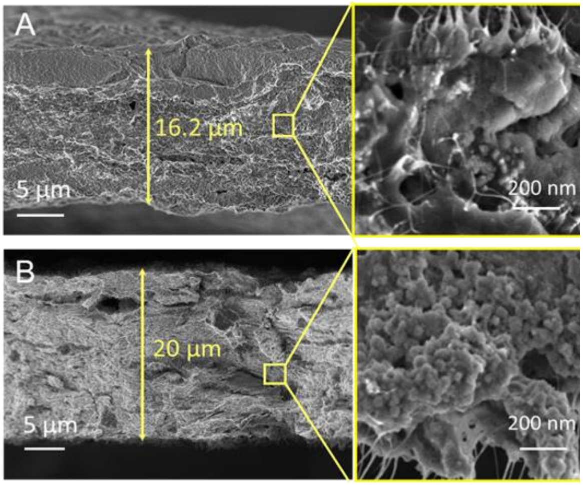

Figure S7. Cross sectional SEM images of the Co_3O_4/SWCNT composite electrode after 200 cycles (a) Li-ion battery anode and (b) Na-ion battery anode. Cross section SEM image of the Co_3O_4/SWCNT composite electrode after 200 cycles (a) Li-ion battery anode and (b) Na-ion battery anode with a high magnified image.

Table S1. The literature comparison of the Co_3O_4 nanostructures for both Li-ion and Na-ion battery anodes.

Ref	Electrode Material	Capacity/Cycle (mAh/g)	Current density (mA/g)
	As Li-ion battery anode		
4	2D holey Co_3O_4 nanosheets	1324/50	100
5	2D porous Co_3O_4 nanosheets	1000/100	400
6	Co_3O_4 nanoparticle film	890/50	45
7	Co_3O_4–graphene networks	630/50	180
8	Co_3O_4 Nanoplates	852/100	500
9	Co_3O_4 Nanoparticles	574.6/50	100
10	Mesoporous nanostructured Co_3O_4	913 /100	100
11	Co_3O_4 /N-doped porous carbon	892/100	100
12	Flower-like Co_3O_4/carbon nanofiber	909/100	200
13	Flower-like Co_3O_4/carbon nanofiber	911/50	200
14	Shale-like Co_3O_4	1045/100	100
15	Peapod-like Co_3O_4@Carbon	862/60	1000
16	Mesoporous Co_3O_4 hollow spheres	700/100	100
17	Co_3O_4 Hollow-structured nanoparticles	770/50	100
18	Co_3O_4/nitrogen modified graphene	900/100	120
19	Porous Co_3O_4 nanoneedle arrays	924/100	50
20	Co_3O_4/graphene	778/42	200
	Co_3O_4/SWCNT composite	1108/10	125
		880/200	625

	As Na-ion battery anode		
4	2D holey nanosheets	566/20	100
21	Co_3O_4 @ stainless steel mesh	509/20	50
22	Co_3O_4 nanosheets	600/10	100
23	Co_3O_4/3D graphene	524/50	25
24	Co_3O_4@CNTs	440/30	25
25	Nanostructured Co_3O_4	447/50	25
26	bowl-like hollow Co_3O_4	300/10	178
	Co_3O_4/SWCNT composite	1083/10	125
		735/200	625

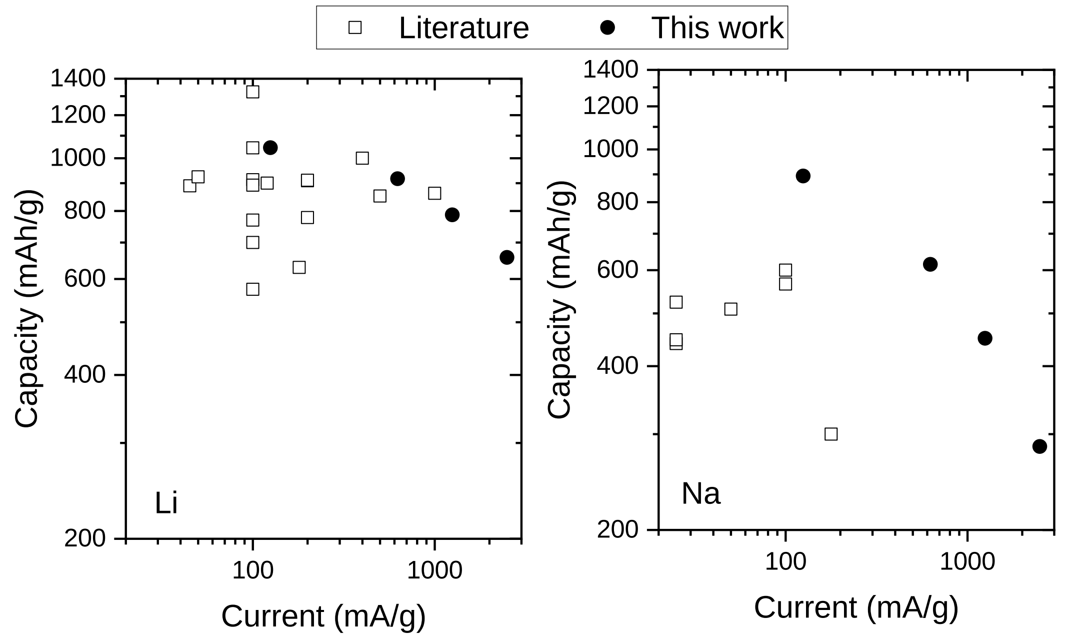

Figure S8. The data in Table S1 presented graphically. This shows our data to be close to the state of the art for LIBs and our SIB data to be beyond state of the art.

S7. Modelling rate performance

The characteristic time associated with charge / discharge, τ, is given by an equation[27] which can be rewritten slightly to highlight the fact that it can be separated into capacitive and diffusive terms.

$$\tau = \tau_C + \tau_D =$$

$$\tau = 28Q_V\left[\frac{L_E^2}{2\sigma_{OOP}} + \frac{L_E^2}{2\sigma_{BL}P_E/\kappa_E} + \frac{L_E L_S}{\sigma_{BL}P_S/\kappa_S}\right] + \left[\frac{L_E^2}{D_{BL}P_E/\kappa_E} + \frac{L_S^2}{D_{BL}P_S/\kappa_S} + \frac{L_{AM}^2}{D_{AM}}\right]$$

Capacitive (resistive) terms   Diffusive terms

We can estimate τ both for both Li-ion and Na-ion electrodes we use known/estimated values of estimate the relevant parameters as given in table S2

[N.B. Here we neglected the time associated with the electrochemical reaction. Here we use the diffusion coefficient in the pores is reduced relative to that in bulk liquid as described by the Bruggeman equation: $D_{Pore} = D_{BL}P/\kappa$, where P is the porosity of the electrode and κ is the tortuosity factor.[28, 29]]

Table S2. Co_3O_4/SWCNT composite electrodes parameters

Parameter	Symbol and value	Comment
Volumetric capacity	Q_V	Found using $Q_V = \rho_E Q_{M,Act}(1-M_f)$
Low-rate specific capacity	$Q_{M,Act}$ =1108 mAh/g (Li)<br>$Q_{M,Act}$ =1083 mAh/g (Na)	From rate fit

Electrode density	ρ_E=960 kg/m^3	-
CNT mass fraction	M_f=0.2	-
Electrode thickness	L_E=10 μm	-
Electrode conductivity	σ_{OOP}=1 S/m	Estimated by comparison with previous results
Bulk electrolyte conductivity	σ_{BL}=0.8 S/m	Similar for both (*J. Electrochem. Soc.*, **2021**, *168* (4), 040538)
Electrode porosity	P_E=0.85	-
Electrode tortuosity	κ_E=2	estimated
Separator thickness	L_S=20 μm (Li)	-
	L_S=350 μm (Na)	-
Separator porosity	P_S=0.4 (Li)	Typical
	P_S=0.9 (Na)	Estimated from product data sheet
Separator tortuosity	κ_S=3 (Li)	Typical
	κ_S=1 (Na)	Estimated due to high porosity
Bulk electrolyte diffusivity	3×10^{-10} m^2/s (Li)<br>3×10^{-10} m^2/s (Na)	From *J. Electrochem. Soc.*, **2021**, *168* (4), 040538
Solid state diffusion length	L_{AM}=50 nm	Estimated from platelet thickness

Solid state diffusivity	D_{AM}=1.5×10^{-17} m^2/s (Li)<br>D_{AM}=6.5×10^{-18} m^2/s (Na)	***chosen to give value of τ roughly matching measured value.***
**Calculated Capacitive contribution to τ**	**τ_C =44s (Li)**<br>**τ_C =106s (Na)**	**-**<br>**-**
**Calculated Diffusive contribution to τ**	**τ_D = 177s (Li)**<br>**τ_D =839s (Na)**	**-**<br>**-**
**Total calculated τ**	**τ = 221s (Li)**<br>**τ = 945s (Na)**	**Experimental: τ = 216s (Li)**<br>**τ = 936s (Na)**
**Estimate n from** $n \sim 0.5 + 0.5 \times \frac{\tau_C}{\tau_C + \tau_D}$	**n= 0.59 (Li)**<br>**n= 0.56 (Na)**	**Experimental: n = 0.54 (Li)**<br>**n = 0.52 (Na)**

References

(1) Barker, A. L.; Unwin, P. R. Measurement of Solute Partitioning across Liquid/Liquid Interfaces Using Scanning Electrochemical Microscopy−Double Potential Step Chronoamperometry (SECM−DPSC): Principles, Theory, and Application to Ferrocenium Ion Transfer Across the 1,2-Dichloroethane/Aqueous Interface. *J. Phys. Chem. B* **2001**, *105* (48), 12019-12031. DOI: 10.1021/jp010591v.

(2) Kaur, H.; Konkena, B.; Gabbett, C.; Smith, R.; McCrystall, M.; Tian, R.; Roy, A.; Carey, T.; Vega-Mayoral, V.; Nicolosi, V.; et al. Amorphous 2D-Nanoplatelets of Red Phosphorus Obtained by Liquid-Phase Exfoliation Yield High Areal Capacity Na-Ion Battery Anodes. *Adv. Energy Mater.* **2023**, *13* (6), 2203013. DOI: https://doi.org/10.1002/aenm.202203013.

(3) Konkena, B.; Kaur, H.; Tian, R.; Gabbett, C.; McCrystall, M.; Horvath, D. V.; Synnatschke, K.; Roy, A.; Smith, R.; Nicolosi, V.; et al. Liquid Processing of Interfacially

Grown Iron-Oxide Flowers into 2D-Platelets Yields Lithium-Ion Battery Anodes with Capacities of Twice the Theoretical Value. *Small* **2022**, *18* (39), 2203918. DOI: https://doi.org/10.1002/smll.202203918.

(4) Chen, D.; Peng, L.; Yuan, Y.; Zhu, Y.; Fang, Z.; Yan, C.; Chen, G.; Shahbazian-Yassar, R.; Lu, J.; Amine, K.; et al. Two-Dimensional Holey Co_3O_4 Nanosheets for High-Rate Alkali-Ion Batteries: From Rational Synthesis to in Situ Probing. *Nano Letters* **2017**, *17* (6), 3907-3913. DOI: 10.1021/acs.nanolett.7b01485.

(5) Li, L.; Jiang, G.; Sun, R.; Cao, B. Two-dimensional Porous Co_3O_4 Nanosheets for High-performance Lithium Ion Batteries. *New J. Chem.* **2017**, *41* (24), 15283-15288, DOI: 10.1039/C7NJ03415F.

(6) Ha, D.-H.; Islam, M. A.; Robinson, R. D. Binder-Free and Carbon-Free Nanoparticle Batteries: A Method for Nanoparticle Electrodes without Polymeric Binders or Carbon Black. *Nano Letters* **2012**, *12* (10), 5122-5130. DOI: 10.1021/nl3019559.

(7) Sun, H.; Liu, Y.; Yu, Y.; Ahmad, M.; Nan, D.; Zhu, J. Mesoporous Co_3O_4 Nanosheets 3D Graphene Networks Hybrid Materials for High-performance Lithium Ion Batteries. *Electrochim. Acta* **2014**, *118*, 1-9. DOI: https://doi.org/10.1016/j.electacta.2013.11.181.

(8) Guo, J.; Jiang, B.; Zhang, X.; Tang, L.; Wen, Y.-h. Topochemical Transformation of Co(ii) coordination Polymers to Co_3O_4 Nanoplates for High-performance Lithium Storage. *J. Mater. Chem. A* **2015**, *3* (5), 2251-2257, DOI: 10.1039/C4TA05041J.

(9) Shi, W.-W.; Zhang, H.; Zheng, X.-Y.; Lou, S.-F.; Hu, B.-W.; Yin, G.-P.; Gao, Y.-Z. Two isomorphous Coordination Polymer-derived Metal Oxides as High-performance Anodes for Lithium-Ion batteries. *New J. Chem.* **2017**, *41* (14), 6187-6194, DOI: 10.1039/C7NJ00540G.

(10) Li, C.; Chen, T.; Xu, W.; Lou, X.; Pan, L.; Chen, Q.; Hu, B. Mesoporous Nanostructured Co_3O_4 Derived from MOF Template: a High-performance Anode Material for Lithium-Ion

Batteries. *Journal of Materials Chemistry A* **2015**, *3* (10), 5585-5591, DOI: 10.1039/C4TA06914E.

(11) Hou, Y.; Li, J.; Wen, Z.; Cui, S.; Yuan, C.; Chen, J. Co_3O_4 Nanoparticles Embedded in Nitrogen-doped Porous Carbon Dodecahedrons with Enhanced Electrochemical Properties for Lithium Storage and Water Splitting. *Nano Energy* **2015**, *12*, 1-8. DOI: https://doi.org/10.1016/j.nanoen.2014.11.043.

(12) Park, S.-H.; Lee, W.-J. Hierarchically Mesoporous Flower-like Cobalt Oxide/Carbon Nanofiber Composites with Shell–Core Structure as Anodes for Lithium Ion Batteries. *Carbon* **2015**, *89*, 197-207. DOI: https://doi.org/10.1016/j.carbon.2015.03.039.

(13) Sun, S.; Zhao, X.; Yang, M.; Wu, L.; Wen, Z.; Shen, X. Hierarchically Ordered Mesoporous Co_3O_4 Materials for High Performance Li-Ion Batteries. *Scientific Reports* **2016**, *6* (1), 19564. DOI: 10.1038/srep19564.

(14) Li, H.-H.; Li, Z.-Y.; Wu, X.-L.; Zhang, L.-L.; Fan, C.-Y.; Wang, H.-F.; Li, X.-Y.; Wang, K.; Sun, H.-Z.; Zhang, J.-P. Shale-like Co_3O_4 for High Performance Lithium/Sodium Ion Batteries. *Journal of Materials Chemistry A* **2016**, *4* (21), 8242-8248, 10.1039/C6TA02417C. DOI: 10.1039/C6TA02417C.

(15) Gu, D.; Li, W.; Wang, F.; Bongard, H.; Spliethoff, B.; Schmidt, W.; Weidenthaler, C.; Xia, Y.; Zhao, D.; Schüth, F. Controllable Synthesis of Mesoporous Peapod-like Co_3O_4@Carbon Nanotube Arrays for High-Performance Lithium-Ion Batteries. *Angewandte Chemie International Edition* **2015**, *54* (24), 7060-7064. DOI: https://doi.org/10.1002/anie.201501475.

(16) Sun, H.; Xin, G.; Hu, T.; Yu, M.; Shao, D.; Sun, X.; Lian, J. High-rate Lithiation-induced Reactivation of Mesoporous Hollow Spheres for Long-lived Lithium-Ion Batteries. *Nature Communications* **2014**, *5* (1), 4526. DOI: 10.1038/ncomms5526.

(17) Wang, D.; Yu, Y.; He, H.; Wang, J.; Zhou, W.; Abruña, H. D. Template-Free Synthesis of Hollow-Structured Co_3O_4 Nanoparticles as High-Performance Anodes for Lithium-Ion Batteries. *ACS Nano* **2015**, *9* (2), 1775-1781. DOI: 10.1021/nn506624g.

(18) Lai, L.; Zhu, J.; Li, Z.; Yu, D. Y. W.; Jiang, S.; Cai, X.; Yan, Q.; Lam, Y. M.; Shen, Z.; Lin, J. Co_3O_4/Nitrogen Modified Graphene Electrode as Li-Ion Battery Anode with High Reversible Capacity and Improved Initial Cycle Performance. *Nano Energy* **2014**, *3*, 134-143. DOI: https://doi.org/10.1016/j.nanoen.2013.05.014.

(19) Kong, D.; Luo, J.; Wang, Y.; Ren, W.; Yu, T.; Luo, Y.; Yang, Y.; Cheng, C. Three-Dimensional Co_3O_4@MnO_2 Hierarchical Nanoneedle Arrays: Morphology Control and Electrochemical Energy Storage. *Advanced Functional Materials* **2014**, *24* (24), 3815-3826. DOI: https://doi.org/10.1002/adfm.201304206.

(20) Kim, H.; Seo, D.-H.; Kim, S.-W.; Kim, J.; Kang, K. Highly Reversible Co_3O_4/Graphene Hybrid Anode for Lithium Rechargeable Batteries. *Carbon* **2011**, *49* (1), 326-332. DOI: https://doi.org/10.1016/j.carbon.2010.09.033.

(21) Dou, Y.; Wang, Y.; Tian, D.; Xu, J.; Zhang, Z.; Liu, Q.; Ruan, B.; Ma, J.; Sun, Z.; Dou, S. X. Atomically Thin Co_3O_4 Nanosheet-coated Stainless Steel Mesh with Enhanced Capacitive Na^+ Storage for High-performance Sodium-Ion Batteries. *2D Materials* **2017**, *4* (1), 015022. DOI: 10.1088/2053-1583/4/1/015022.

(22) Xin, D.; Dai, J.; Liu, J.; Wang, Q.; Li, W. Mesocrystal Hexagonal Co_3O_4 Nanosheets for High Performance Lithium and Sodium-Ion Batteries. *Mater. Letters* **2017**, *209*, 388-391. DOI: https://doi.org/10.1016/j.matlet.2017.08.048.

(23) Liu, Y.; Cheng, Z.; Sun, H.; Arandiyan, H.; Li, J.; Ahmad, M. Mesoporous Co_3O_4 Sheets/3D Graphene Networks Nanohybrids for High-performance Sodium-Ion Battery anode. *J. Power Sources* **2015**, *273*, 878-884. DOI: https://doi.org/10.1016/j.jpowsour.2014.09.121.

(24) Jian, Z.; Liu, P.; Li, F.; Chen, M.; Zhou, H. Monodispersed Hierarchical Co_3O_4 Spheres Intertwined with Carbon Nanotubes for use as Anode Materials in Sodium-Ion Batteries. *J. Mater. Chem. A* **2014**, *2* (34), 13805-13809, DOI: 10.1039/C4TA02516D.

(25) Rahman, M. M.; Glushenkov, A. M.; Ramireddy, T.; Chen, Y. Electrochemical Investigation of Sodium Reactivity with Nanostructured Co_3O_4 for Sodium-Ion Batteries. *Chem. Comm.* **2014**, *50* (39), 5057-5060, DOI: 10.1039/C4CC01033G.

(26) Wen, J.-W.; Zhang, D.-W.; Zang, Y.; Sun, X.; Cheng, B.; Ding, C.-X.; Yu, Y.; Chen, C.-H. Li and Na Storage Behavior of Bowl-like Hollow Co_3O_4 Microspheres as an Anode Material for Lithium-Ion and Sodium-Ion Batteries. *Electrochim. Acta* **2014**, *132*, 193-199. DOI: https://doi.org/10.1016/j.electacta.2014.03.139.

(27) Horvath, D. V.; Coelho, J.; Tian, R.; Nicolosi, V.; Coleman, J. N. Quantifying the Dependence of Battery Rate Performance on Electrode Thickness. *ACS Appl. Energy Mater.* **2020**.

(28) Chung, D.-W.; Ebner, M.; Ely, D. R.; Wood, V.; Edwin García, R. Validity of the Bruggeman relation for porous electrodes. *Model. Simul.Mater. Sci. Eng.* **2013**, *21* (7), 074009. DOI: 10.1088/0965-0393/21/7/074009.

(29) Usseglio-Viretta, F. L. E.; Colclasure, A.; Mistry, A. N.; Claver, K. P. Y.; Pouraghajan, F.; Finegan, D. P.; Heenan, T. M. M.; Abraham, D.; Mukherjee, P. P.; Wheeler, D.; et al. Resolving the Discrepancy in Tortuosity Factor Estimation for Li-Ion Battery Electrodes through Micro-Macro Modeling and Experiment. *J. Electrochem. Soc.* **2018**, *165* (14), A3403-A3426. DOI: 10.1149/2.0731814jes.
